# Supplementary material for: Coconut genome assembly enables evolutionary analysis of palms and highlights signaling pathways involved in salt tolerance
Source: Commun Biol. 2021 Jan 22;4:105. doi: 10.1038/s42003-020-01593-x (PMC7822834; doi:10.1038/s42003-020-01593-x)
Supplement: Supplementary file 2 — Supplementary Information [file 42003_2020_1593_MOESM2_ESM.pdf]

Coconut genome assembly enables evolutionary analysis of palms and highlights signaling pathways involved in salt tolerance.

Yaodong Yang, Stéphanie Bocs, Haikuo Fan, Alix Armero, Luc Baudouin, Pengwei Xu, Junyang Xu, Dominique This, Chantal Hamelin, Amjad Iqbal, Rashad Qadri, Lixia Zhou, Jing Li, Yi Wu, Zilong Ma, Auguste Emmanuel Issali, Ronan Rivallan, Na Liu, Wei Xia, Ming Peng, Yong Xiao

Supplementary information

Contents

1. Assessment of linkage map and assembly **3**

Figure S1: Histogram of the protein length in HAIT and in CATD. 3

Figure S2: Dot plot of the linkage between loci of the 16 linkage groups 4

Figure S3: Recombination, gene and marker landscapes 5

Figure S4: Comparison between HAIT linkage mapping and CATD Hi-C mapping 13

2. palm comparative genomics **14**

Figure S5: Coconut paralogy 14

Figure S6: Mapping of coconut duplicated genes onto the putative pre-p ancestor 15

Figure S7: Synteny between coconut and oil palm 16

3. Gene regulation in coconut leaves during salt stress **17**

Supplementary Note 1 17

Figure S8: Signaling pathways in coconut leaves during salt-water stress 19

Figure S9a: 1st animated fig: RNA-Seq expression at 4 h during salt-water stress 20

Figure S9b: 2nd animated fig: RNA-Seq expression at 6 d during salt-water stress 21

Figure S9c: 3rd animated fig: RNA-Seq expression at 10 d during salt-water stress 22

Figure S10: MT3 gene family (ROS signalling pathway) 23

Figure S11: Expression of the ABA-ABAi pathway in coconut leaves under salt stress 24

Figure S11: Expression of the ABA-ABAi pathway in coconut leaves under salt stress 25

4. GBS processing pipeline **27**

Figure S12: Tassel 5 GBS v2 Pipeline 27

Table S1: Parameters used with TASSEL 28

Table S2: Parameters used with Process reseq 29

Table S3: Parameters used with VCF filter 29

Table S4: Parameters used with Tassel_to_J and Joinmap 29

Table S5: Parameters used with Scaffhunter 30

5. RNA-Seq analysis bioinformatics pipeline **31**

Figure S13: RNA-Seq analysis bioinformatics workflow 31

6. REFERENCES **32**

1. Assessment of linkage map and assembly

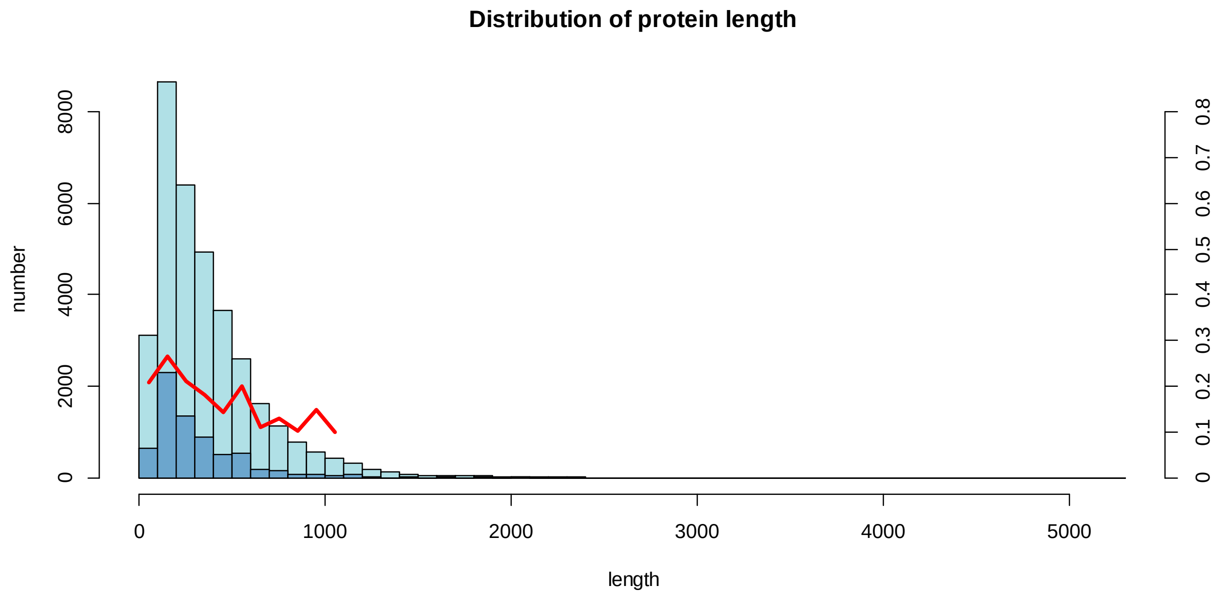

Figure S1: Histogram of the protein length in HAIT and in CATD.

Bin size = 100 bp. The height of the bars is the bars is the number of proteins in the CATD (C), the pale blue segment is the number of proteins in the HAIT (H). The dark blue segments represent the additional proteins in CATD. The red line is D=(C-H)/C for length <1,200 (scale to the right). CATD tend to have more proteins of small size but the effect of size is limited (+25% around 100 bp, +10% around 1,000).

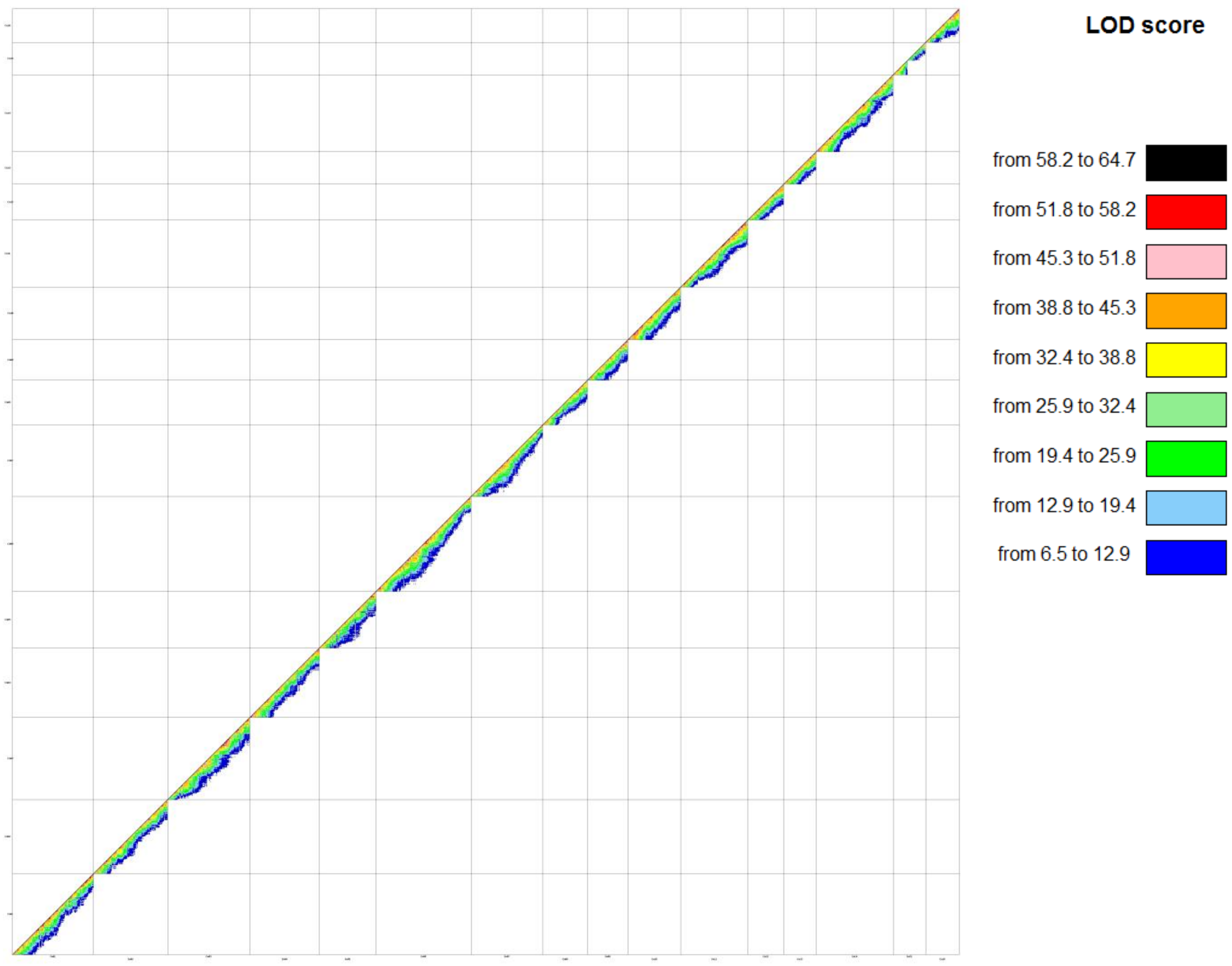

Figure S2: Dot plot of the linkage between loci of the 16 linkage groups

Figure S3: Recombination, gene and marker landscapes

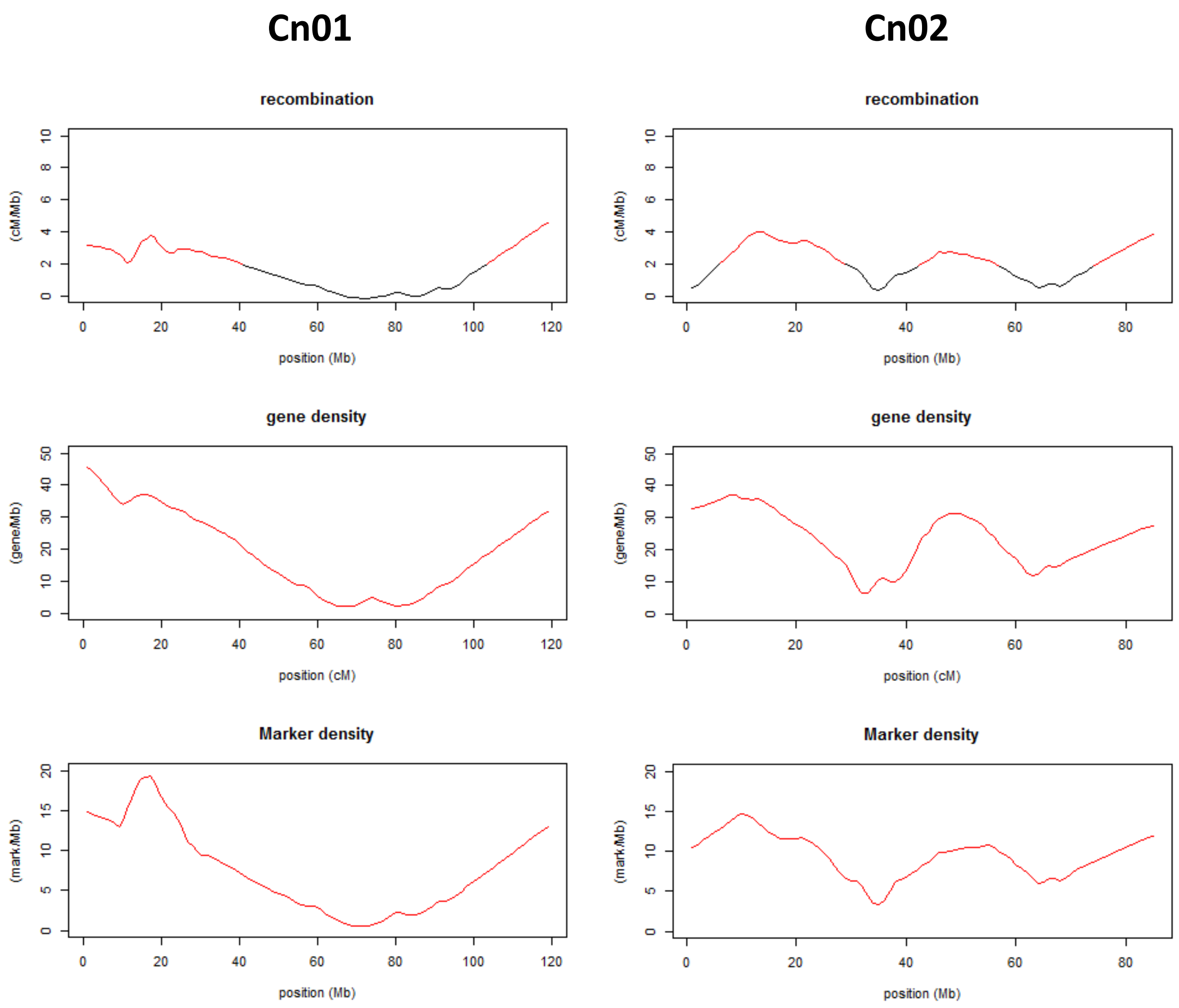

Figure S3 continued

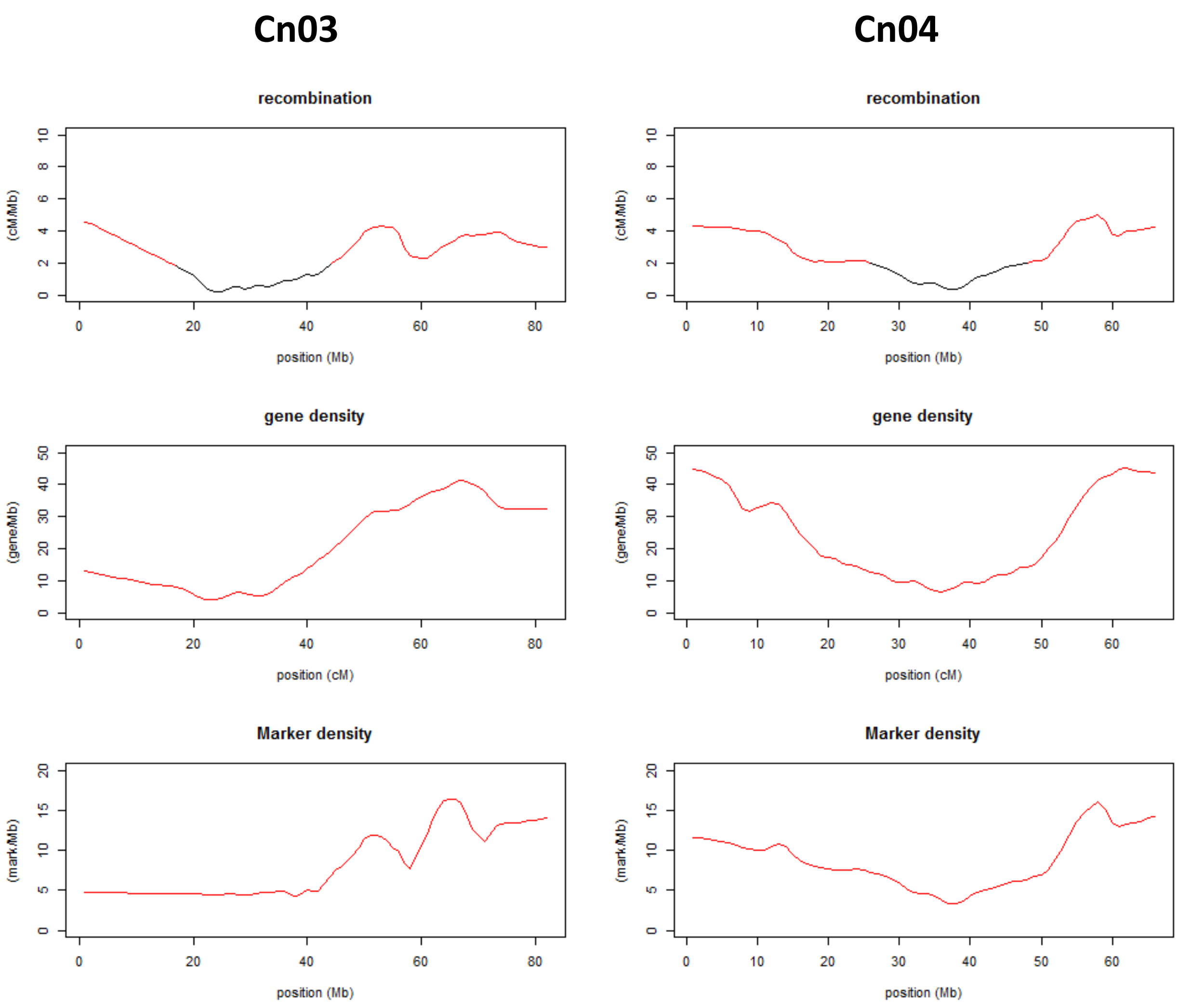

Figure S3 continued

Cn05

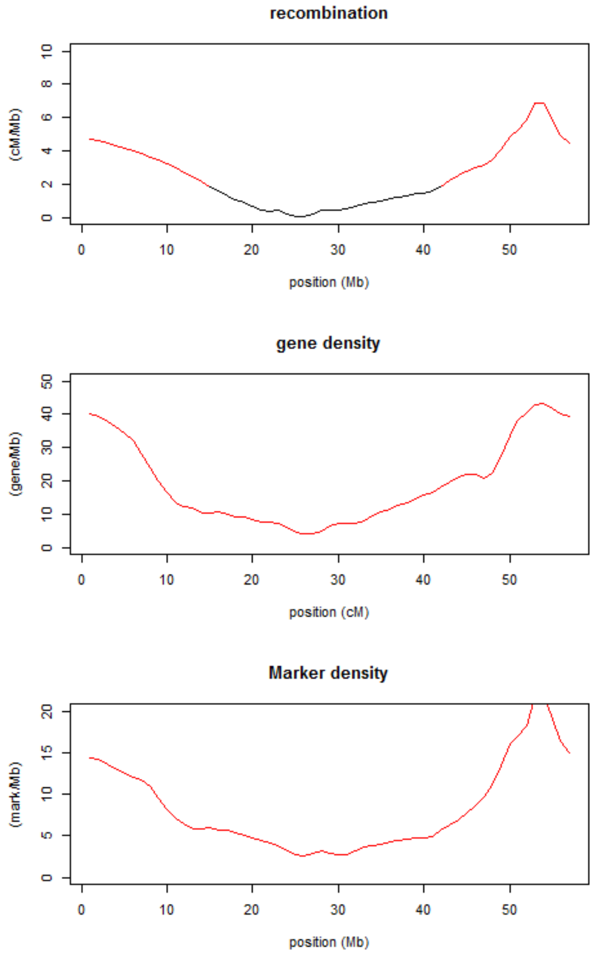

Cn06

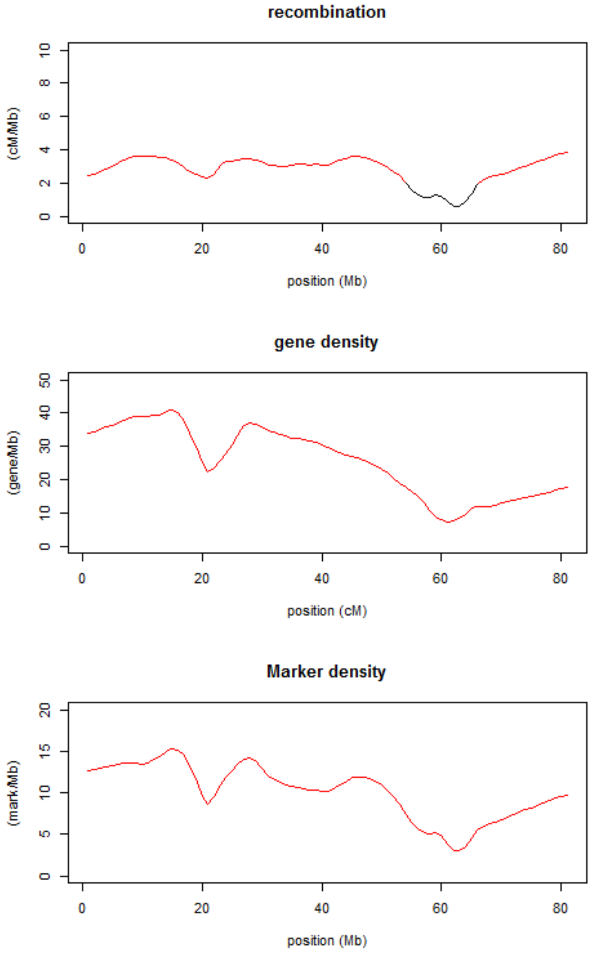

Figure S3 continued

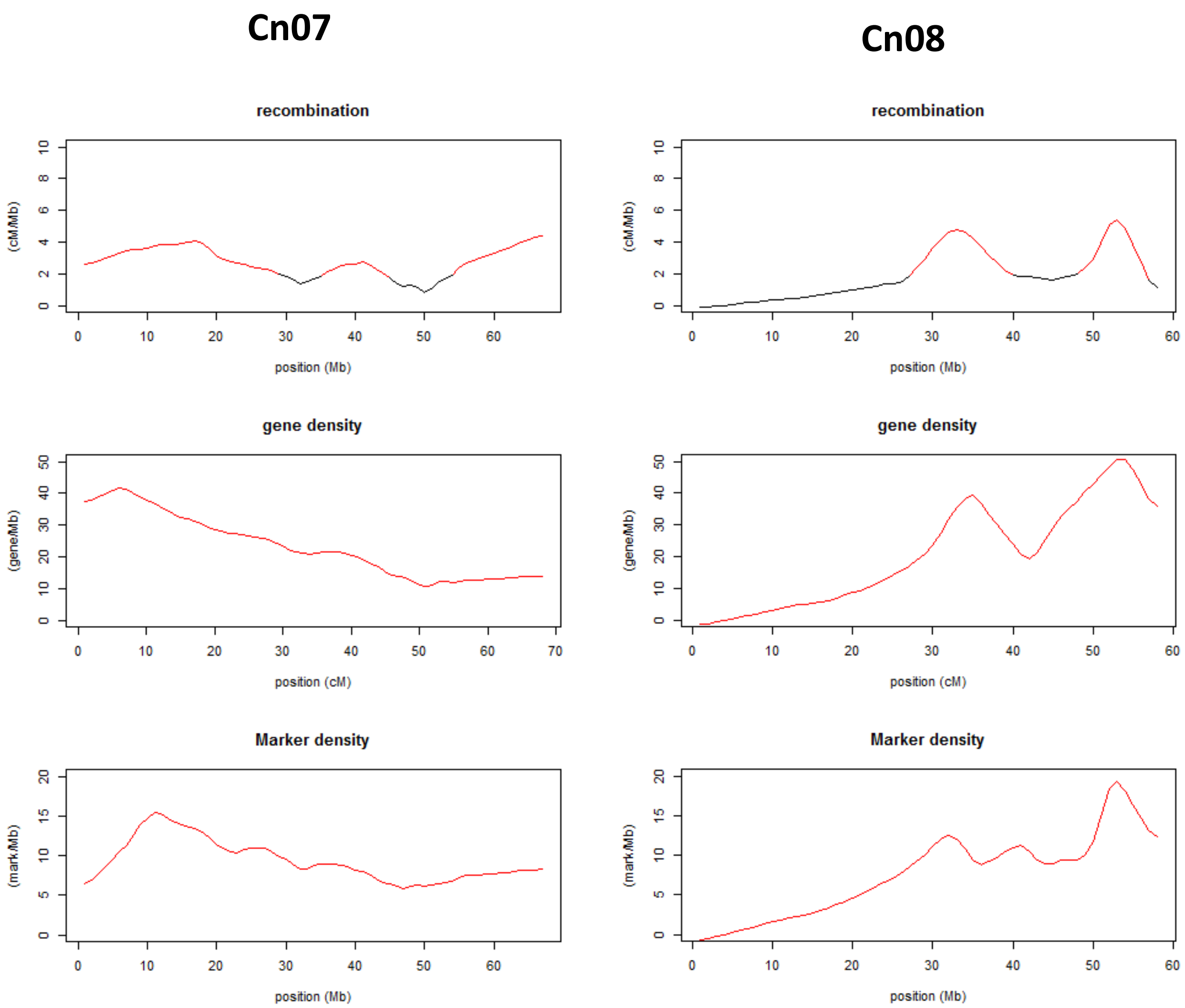

Figure S3 continued

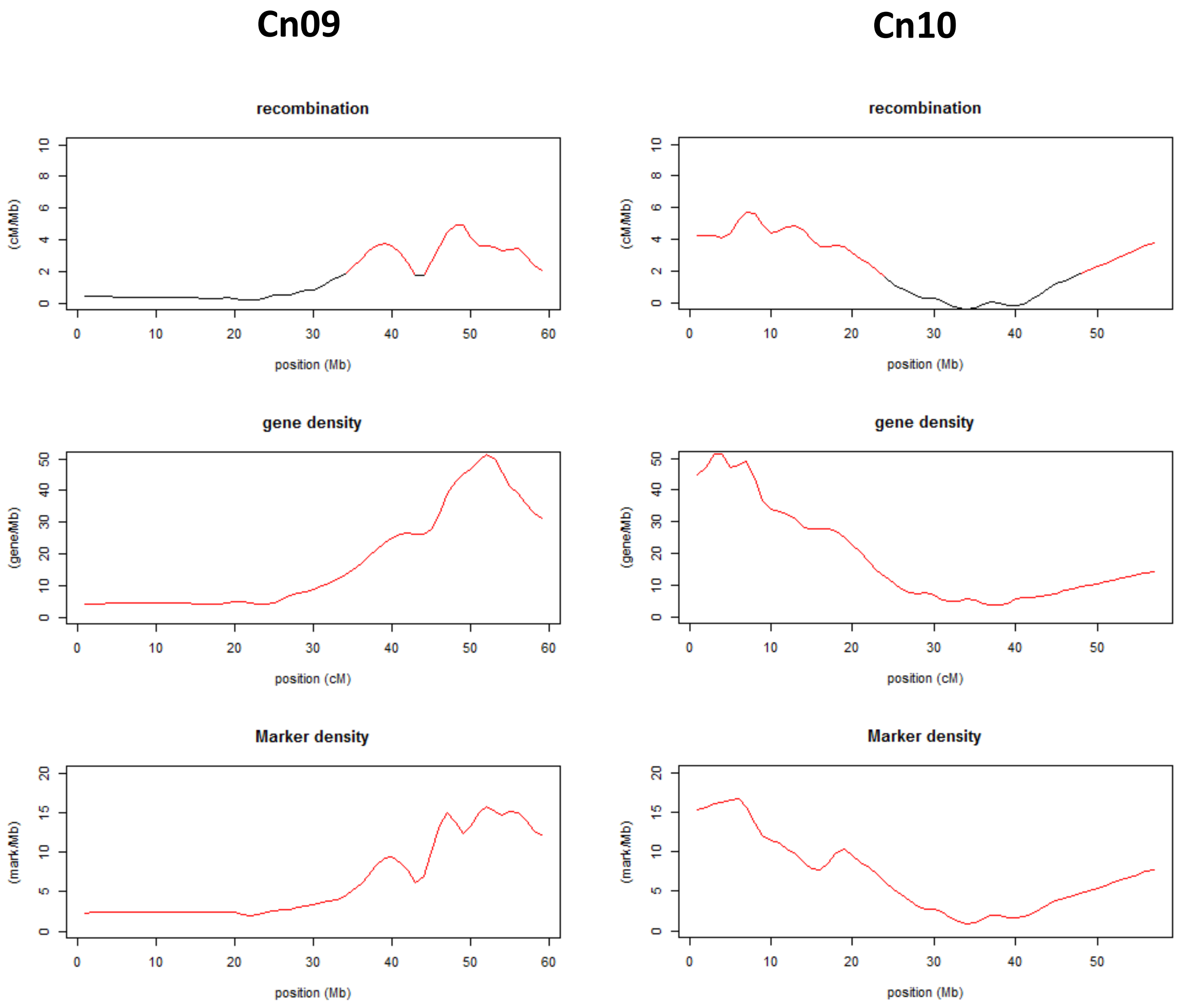

Figure S3 continued

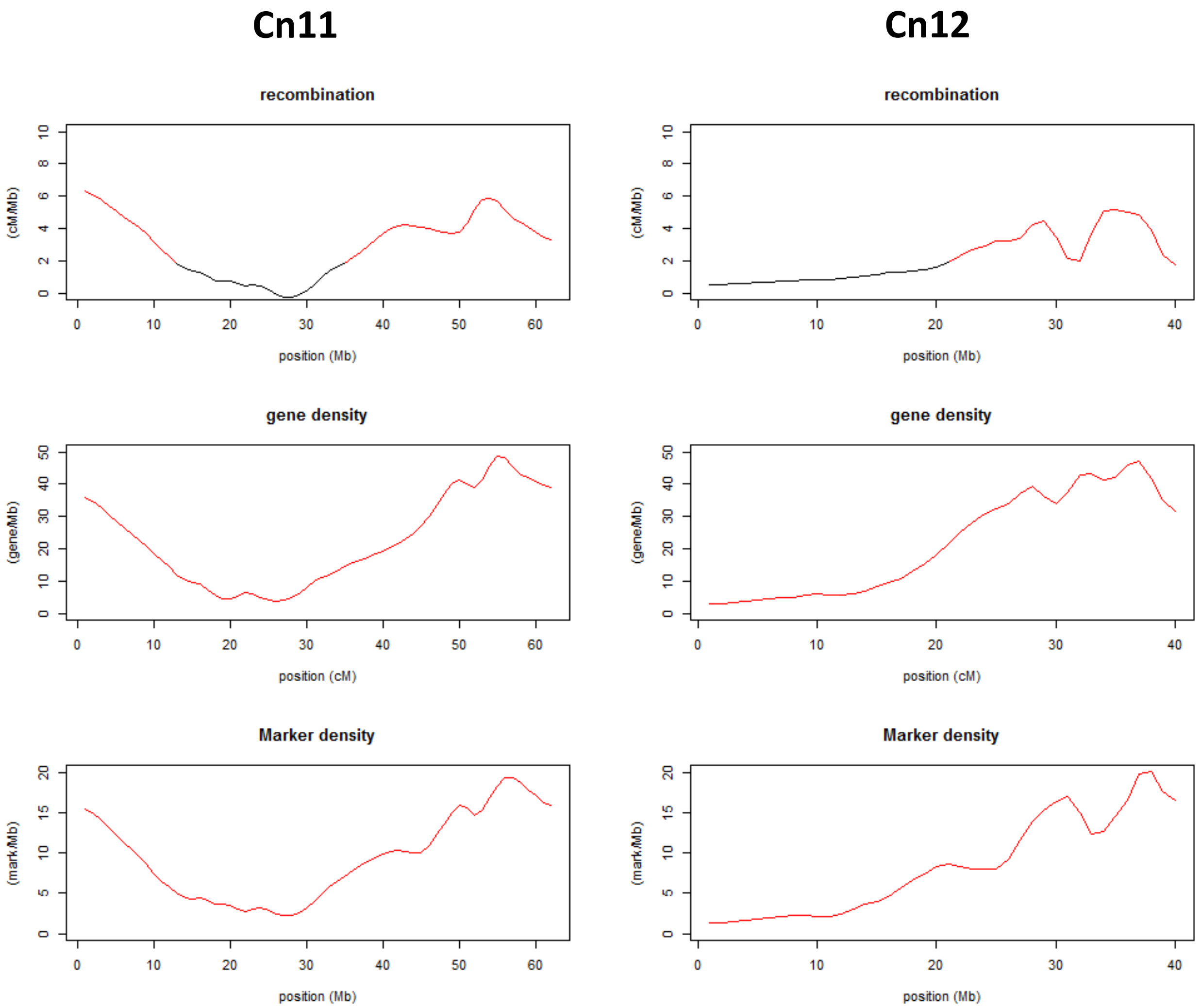

Figure S3 continued

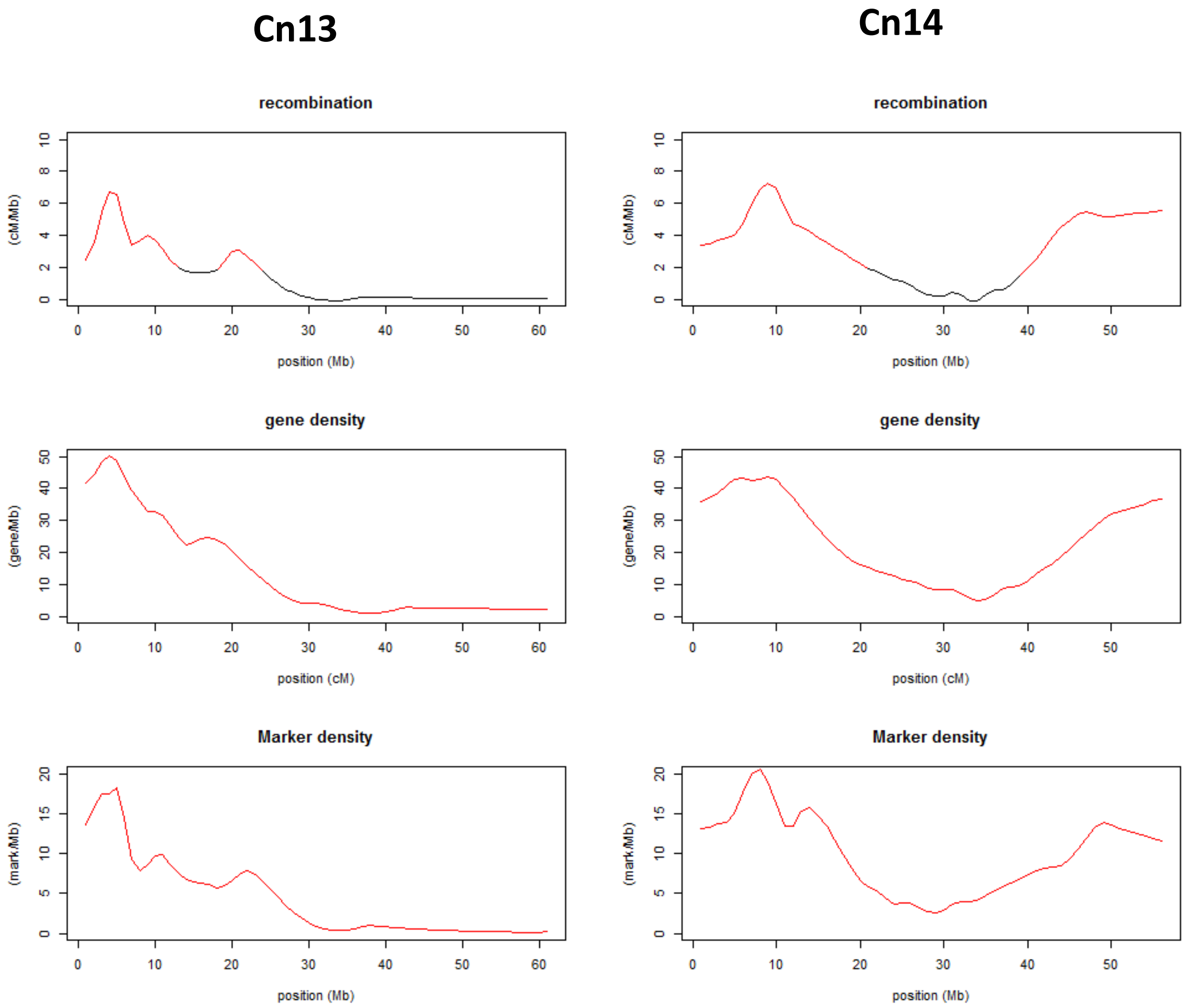

Figure S3 continued

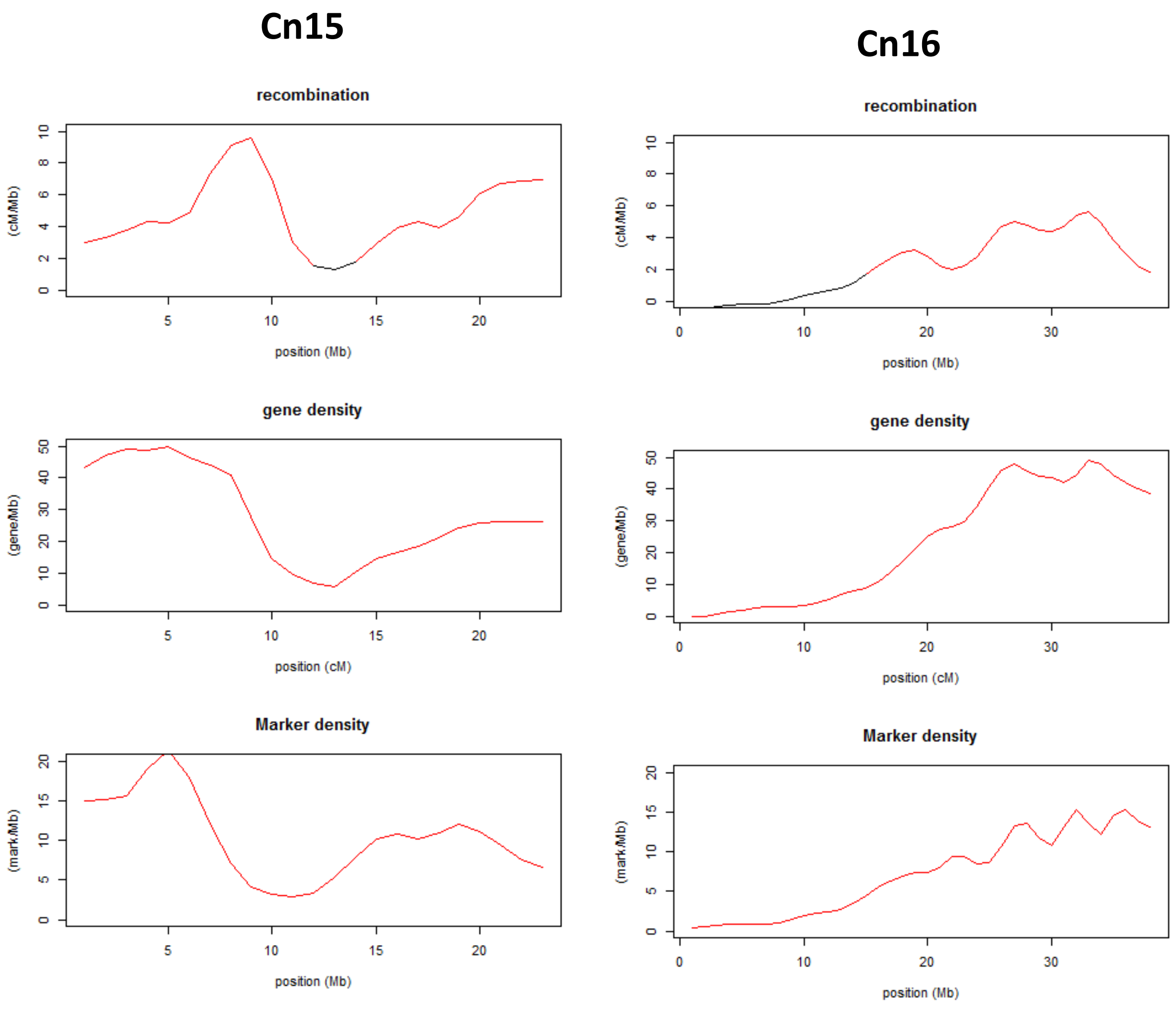

Figure S3: Recombination, gene and marker landscapes

The 16 figures below consists in three panels per linkage group, respectively the recombination rate (in cM/Mb), the density of genes and marker density. Regions with less than 2 cM/Mb (marked in black in the first panel) correspond approximately to heterochromatin, have fewer genes and markers than euchromatin and are likely to be underrepresented in the current version of the sequence. Note the presence of deep “valleys” around 60 Mb on Cn02 and around 40 Mb on Cn08, corresponding to chromosome fusions occurring after divergence from oil palm. Note that, due to incomplete assembly in heterochromatin, values tend to be overestimated in these regions.

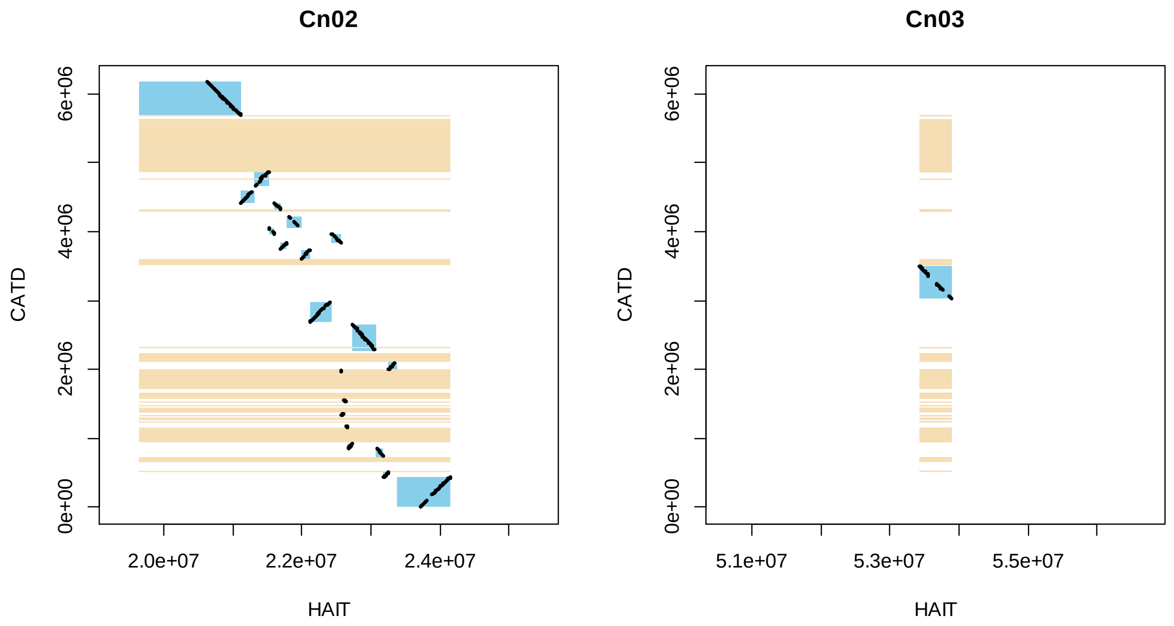

Figure S4: Comparison between HAIT linkage mapping and CATD Hi-C mapping

Matches between one of the largest scaffolds of the CATD sequence (cultivar Catigan Green Dwarf; D0, y axis) and pseudomolecules produced by linkage mapping on HAIT (coconut cultivar Hainan Tall; x axis) are represented by black dots. The blue squares correspond to anchored HAIT scaffolds (two of them extend beyond D0). Most of them belong to Cn02, but the Hi-C mapping also recruited one scaffold from Cn03, thus creating a chimeric scaffold. The orders of scaffolds on Cn02 and on D0 are similar but not identical. This could be due in part to statistical uncertainty in recombination rates. A large number of small unassembled HAIT scaffold match on D0 and are represented by beige rectangles.

2. palm comparative genomics

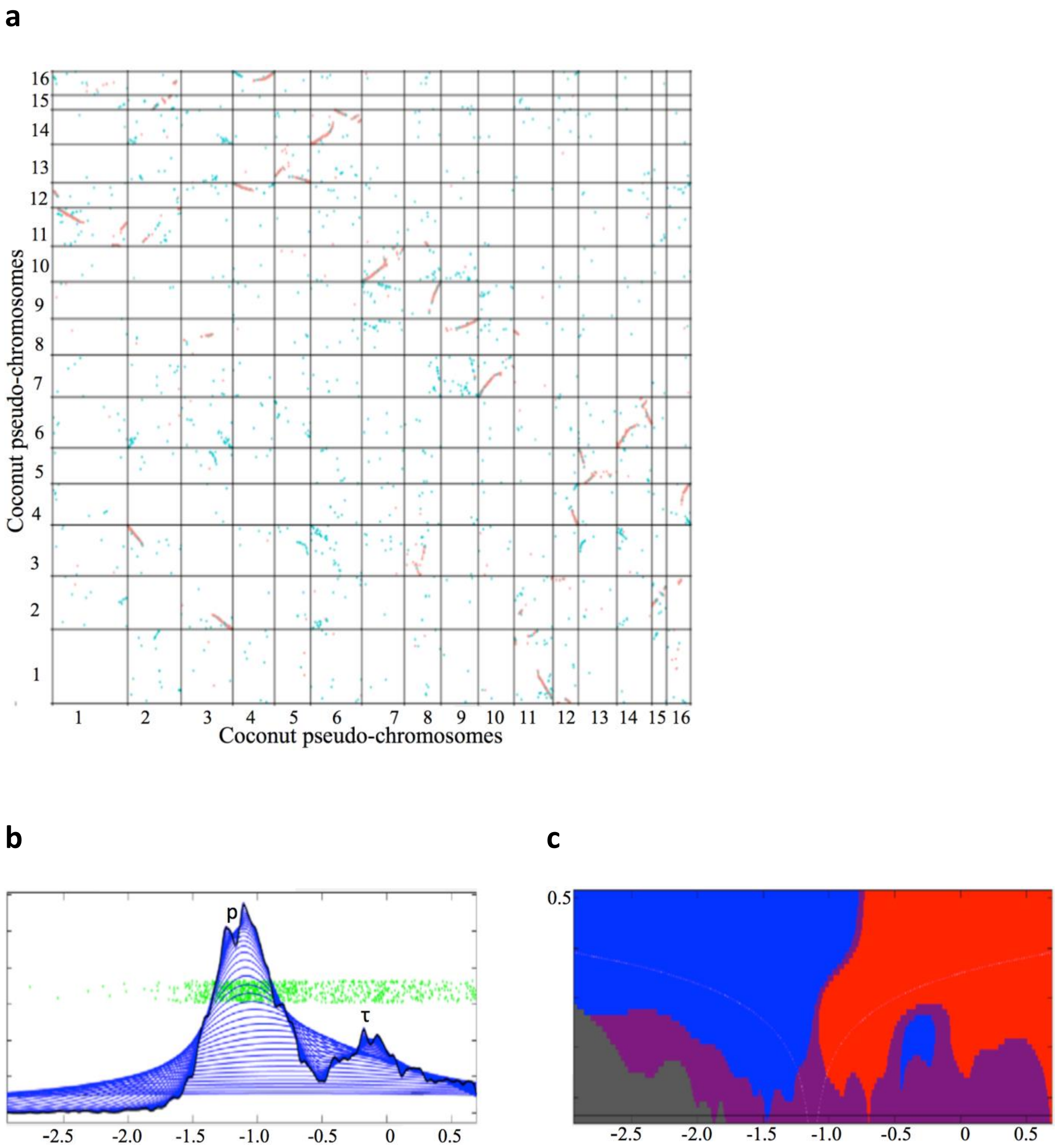

Figure S5: Coconut paralogy

(a) Relations of syntenic paralogy between coconut polypeptides. The red dots are pairs of paralog products of the *p* WGD, while the blue dots represent the *τ* whole genome duplication (WGD).

(b) Distribution of the natural logarithm of dS of aligned CDS pair. The two peaks represent the two WGDs.

(c) Sizer map of log dS distribution. The blue zones represent significant increases in slope, red areas significant decrease and areas purple slopes close to zero.

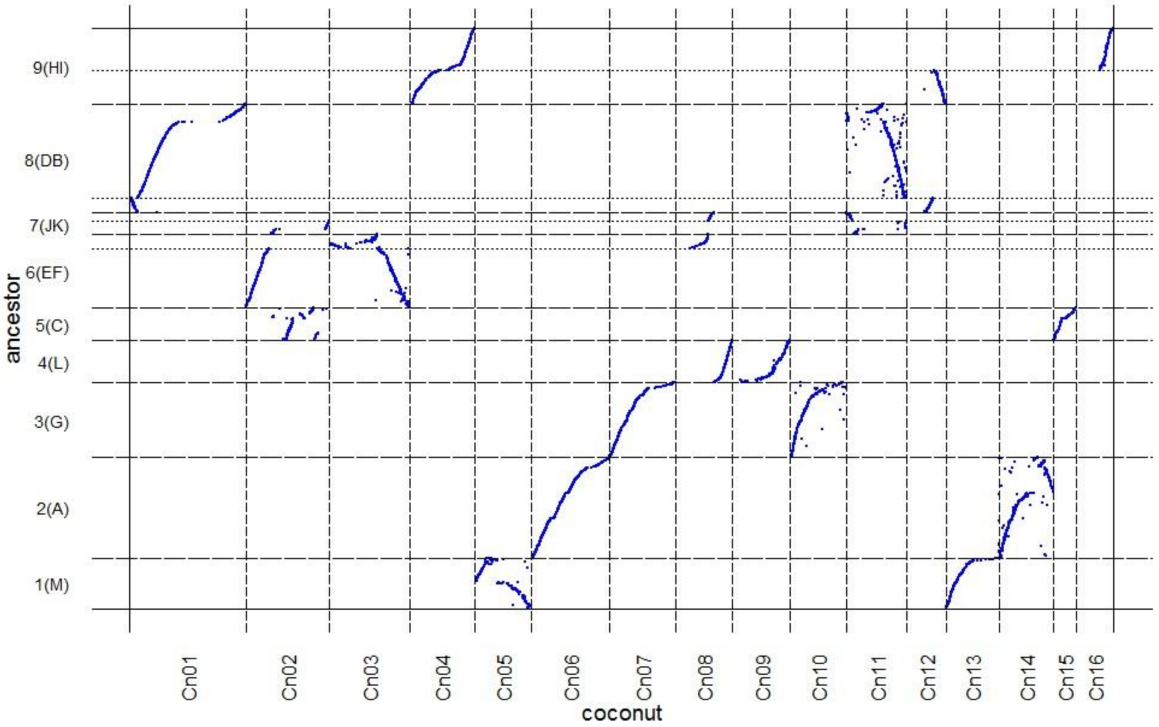

Figure S6: Mapping of coconut duplicated genes onto 9 chromosomes of the putative pre-p ancestor
The synteny blocs can be identified by referring to figure 4 (E.g. block B is shared between Cn01 and Cn11).

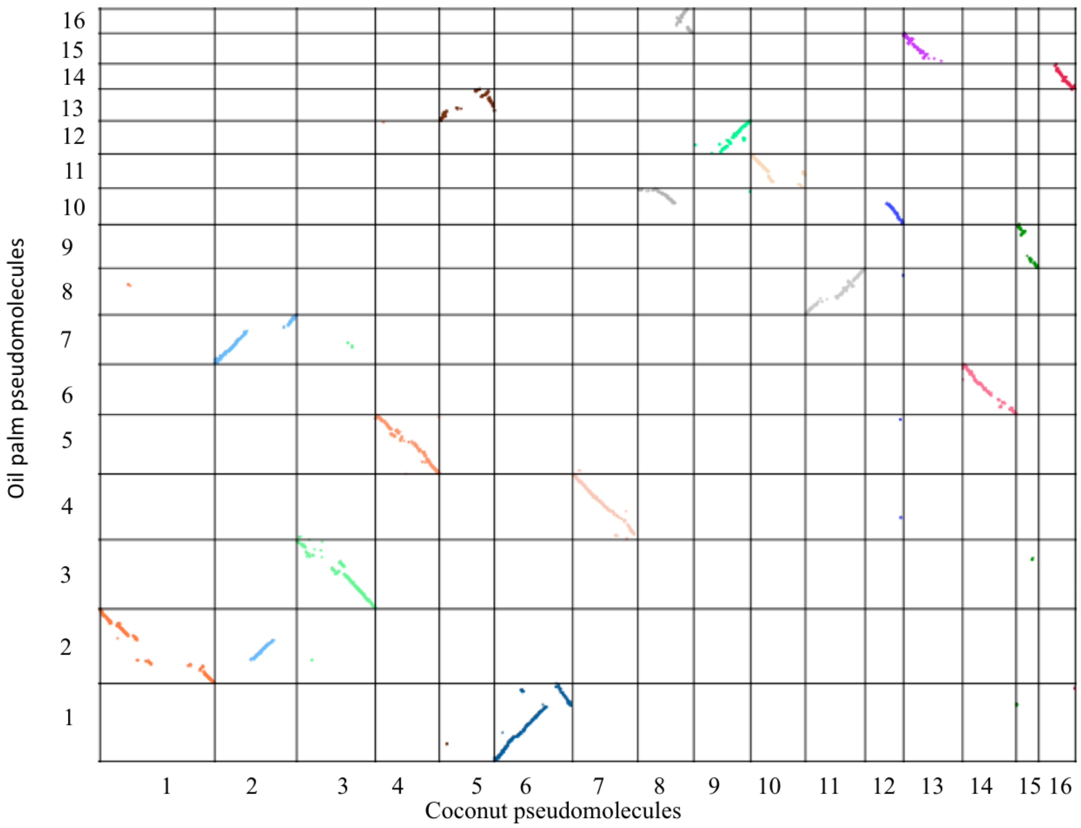

Figure S7: Synteny between coconut and oil palm

Relations of syntenic orthology between *Elaeis guineensis* and *Cocos nucifera* polypeptides. Each dot corresponds to a pair of orthologous protein sequences. The dots are painted according the coconut chromosomes.

3. Gene regulation in coconut leaves during salt stress

Supplementary Note 1

We provide a synthetic representation of the most important early signaling pathways in Figure S8. Figure S9 represents the evolution of gene expression after 4 hours (S9a), 6 days (S9b) and 10 days (S9c).

Our results, shown in Figure S9, confirmed that coconut leaves were under stress because, out of the four protective effectors considered as salt stress markers in rice by Soda et al.[1]. Transcripts of two stress markers genes were differentially expressed at least at the late stage: metallothionein MT3 with expression class number (ECN) 62 and grey stars on Figure 6 and S10, and a dehydrin (DHN), of the Late Embryogenesis Abundant (LEA) family with ECN 13 and a grey star on Figure S11. Another dehydrin ERD10 (early response to dehydration with ECN 16 and grey stars on Figure S11) exhibited the same tendency although below the significance threshold. Regarding the fourth one, Glutathione S-transferase (GST), ECN 35, grey stars on Figure 6), three copies were CHE. Constitutive high expression of GSTs could be interpreted as a coconut halophytic feature as suggested in previous papers[2,3].

As a complement to the results given in the article, we focus here on key players of the abscisic acid (ABA) dependent early signaling pathway (Figure S11). A decrease of PP2C (Protein phosphatase 2C with ECN 2 and yellow stars in Figures S11a and S11c) could enhance ABA signaling which triggers several responses such as expression regulation of transcription factors involved in synthesis of protective molecules (*e.g*. CAPRICE, or CPC with ECN 5 and a yellow star on Figure S11d) or in flowering time (*e.g.* CONSTANS-LIKE, or COL with ECN 4 and yellow stars on Figure S11e) and thus contributes to the osmotic homeostasis[4].

Among the studied players of the Ca^{2+}/PLC signaling pathway, none where observed differentially expressed at the early stage (CN01_scaffold078790G000010 PLD ECN 37 was observed differentially expressed only during the late stage; Supplementary Data 3).

Regarding reciprocal **crosstalks** (coloured arrows) between ROS and ABA signaling pathways during salt-water shock in coconut leaves, RBOHF activated by Ca^{2+} produces ROS; among which, H_2O_2, activates the ABA biosynthesis[6] and deactivates PP2C[9] that was already repressed by ABA. In turn, SNRK2 activates RBOHF[6] and stomatal closure triggered by ABA compromises photosynthesis with H_2O_2 production in the chloroplast.

The most striking observations about transcriptional regulation of key players 4h after the salt-water shock are:

PP2C hub repressor gene is underexpressed. On top of it, H_2O_2[9] inactivates it. Both effects tend to overexpress several ABA early signalling pathway players such as the MYB CPC transcription factor. This contributes to enhance ABA biosynthesis at a later stage along with Ca^{2+} and H_2O_2[6].

We also note a transcriptional overexpression of SODC, activator of ROS early signalling. Other factors are likely to enhance this signalling pathway: RBOH activation by Ca^{2+}, CIPK26, SNRK2[6], photosynthesis reduction due to stomatal closure[10].

Regarding ionic stress signaling, PM-AKT (orthologous to AKT2_ARATH) could be the sodium sensor[11,12] that activates the Ca^{2+}/PLC signaling that activates in turn the SOS or CAM signaling[13] (being under both regulations: osmotic with calcium signature and ionic for Na^{+} signature) that activates and induces bHLH and AKT2 that adjusts the Na^{+}/K^{+} ratio to keep it low and triggers stomatal opening.

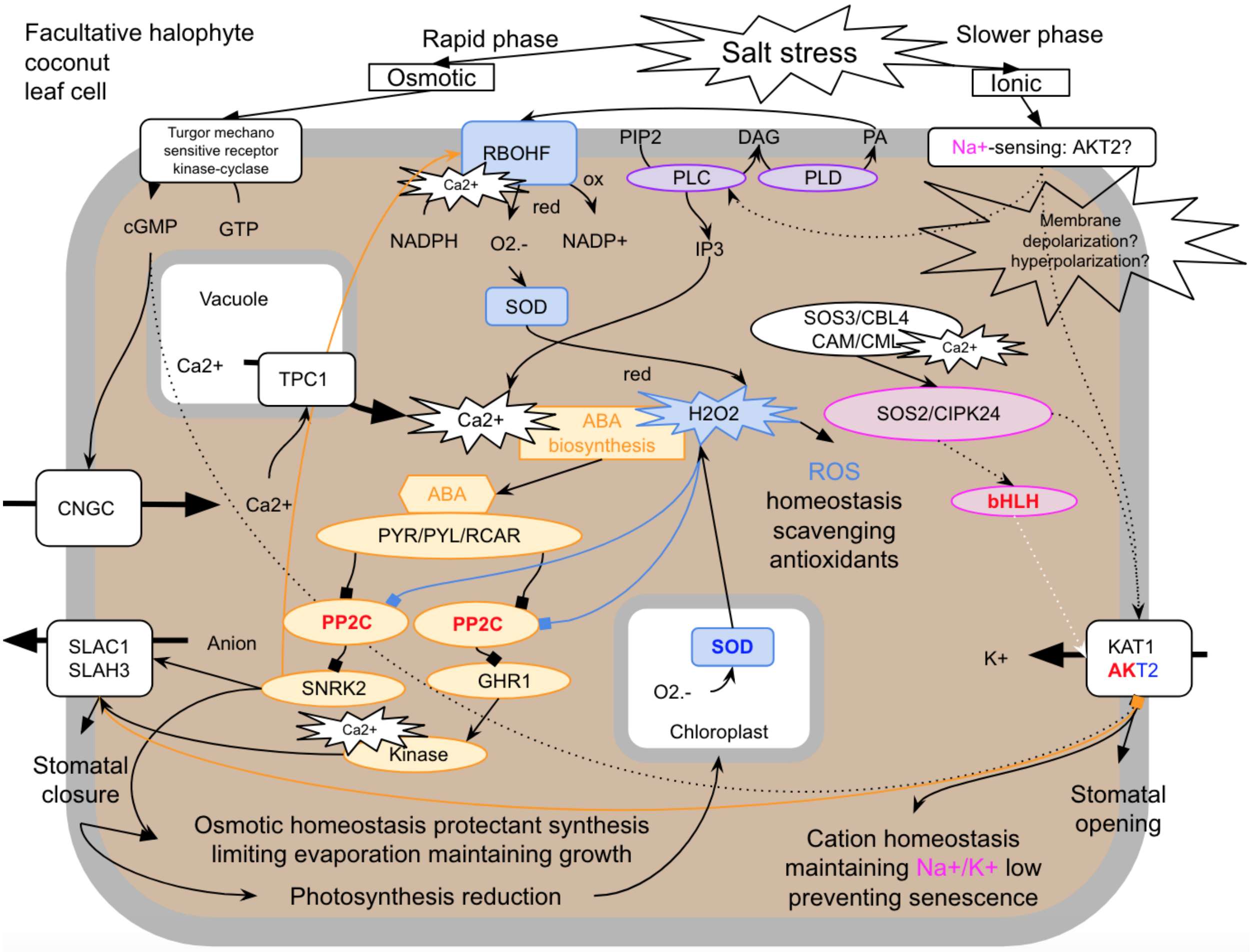

Figure S8: Signaling pathways in coconut leaves during salt-water stress

Four main pathways are modelled based on previous studies[4-8] to signal osmotic stress with ABA dependent (golden yellow) and ionic stress Ca^{2+}/PLC (purple) and SOS (magenta). ROS (blue) seems to be a crossroad pathway. Ca^{2+} and H_2O_2 could be the main signaling molecules. The polypeptides drawn in the shape of (i) a vertical oval represents sensors (e.g. membrane receptors); (ii) a horizontal oval represents signaling molecules such a MAPK cascade, transcription factors, ABA receptors; (iii) a filled rectangle represent inducible transcription activators and stress tolerance effectors and (iv) empty rectangle are phytohormones. Hypothesized links are illustrated with dashed lines. The lines illustrate pathways that are not shown and described in detail. Arrows represent up-regulation of transcriptional expression or activation whereas lines ended with a small square represent down-regulation of transcriptional expression or inhibition. The gene symbols in bold mean that they are differentially expressed at an early time (four hours), either upregulated (in blue) or down regulated (in red).

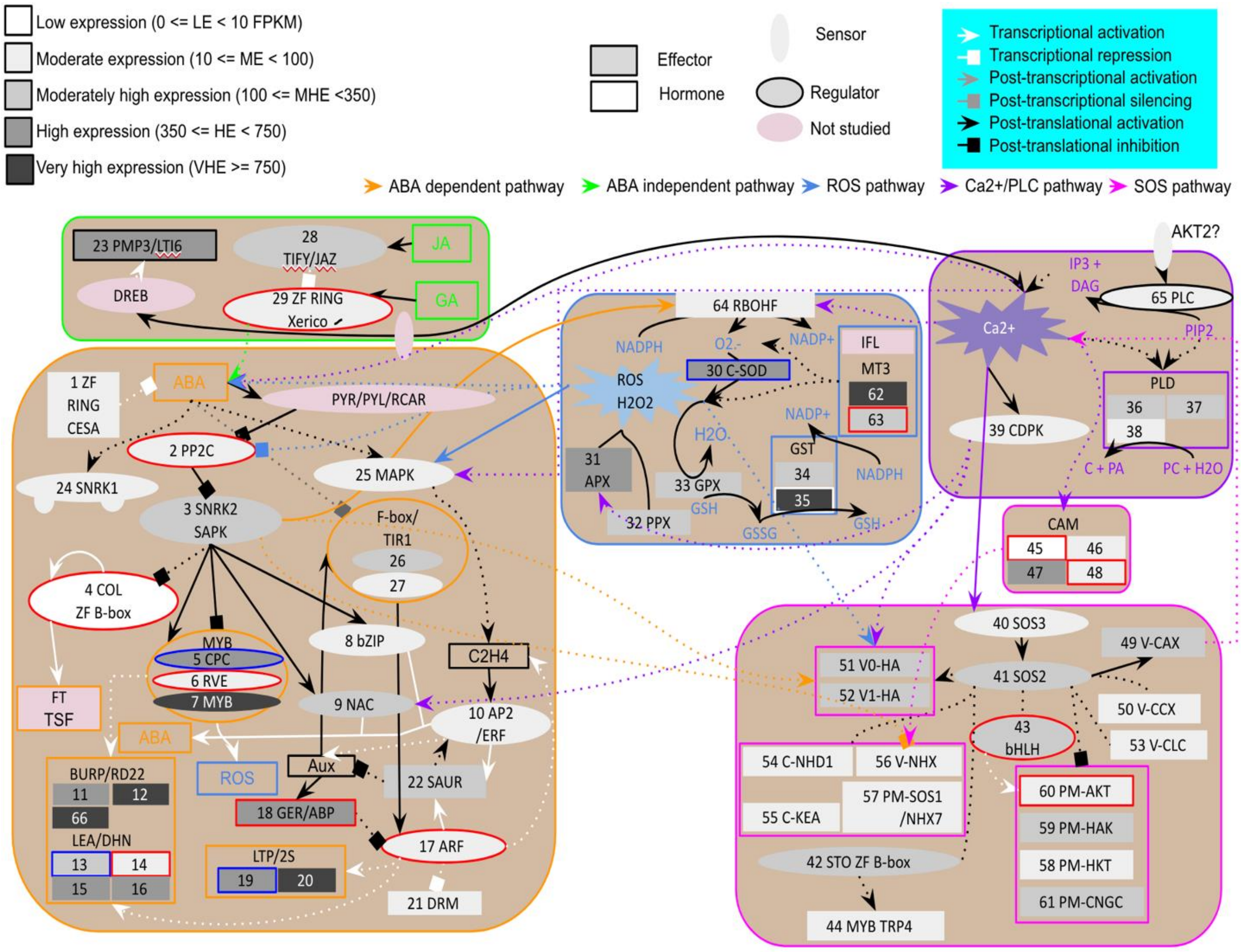

Figure S9a: 1st animated fig: RNA-Seq expression at 4 h in coconut leaf signaling pathways during salt-water stress

Salt stress induces both osmotic and ionic stress signallings tranduced via five main pathways in rice[4], coloured as follow:

1. Abscisic acid dependent pathway (ABA golden yellow),
2. ABA-independent pathway (ABAi in green, e.g. jasmonic acid),
3. Reactive oxygen species pathway (ROS in blue)
4. Cytosolic Ca^{2+} dication spikes induced by the phospholipase pathway (Ca^{2+}/PLC in purple) and
5. Calmodulin and salt overly sensitive pathways (CAM/SOS in magenta).

We assigned the same expression class numbers (ECN) to coconut paralogous genes showing similar expression profiles (cf supplementary data 3). The shade of the box surrounding the ECN and gene code represents the absolute expression level while the border represents its variation. A red or blue border denotes down or up-regulation respectively (relative to T0) while a black or white border denotes constitutively high expression.

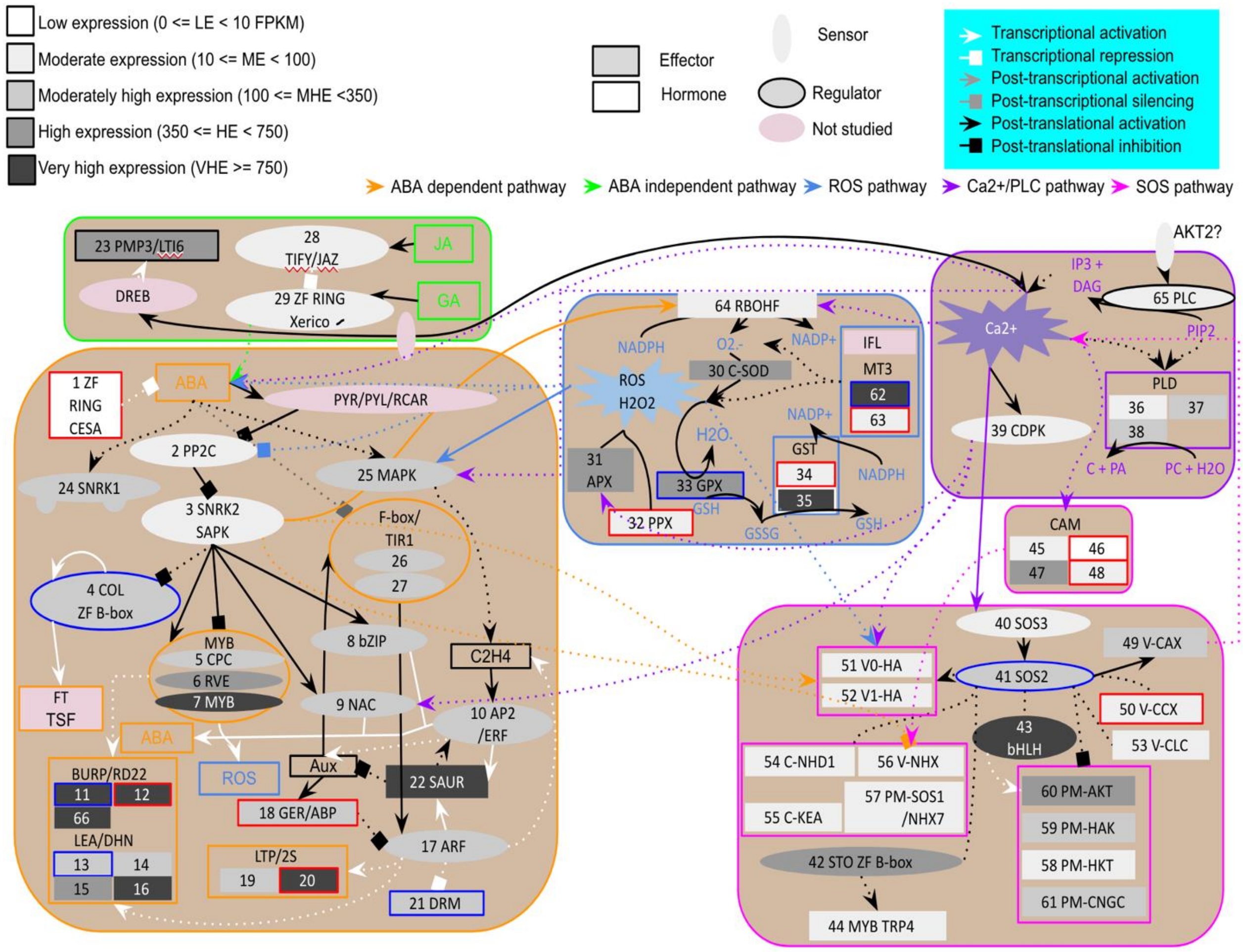

Figure S9b: 2nd animated fig: RNA-Seq expression at 6 d in coconut leaf signaling pathways during salt-water stress

Graphical conventions are the same as in figure S9a. In contrast with the early response, where 12 proteins were under-expressed and four over-expressed, this trend is gradually reversed during the late response: 10 proteins are ten under-expressed ECNs against six over-expressed when the time point 6d is compared to the time point 0.

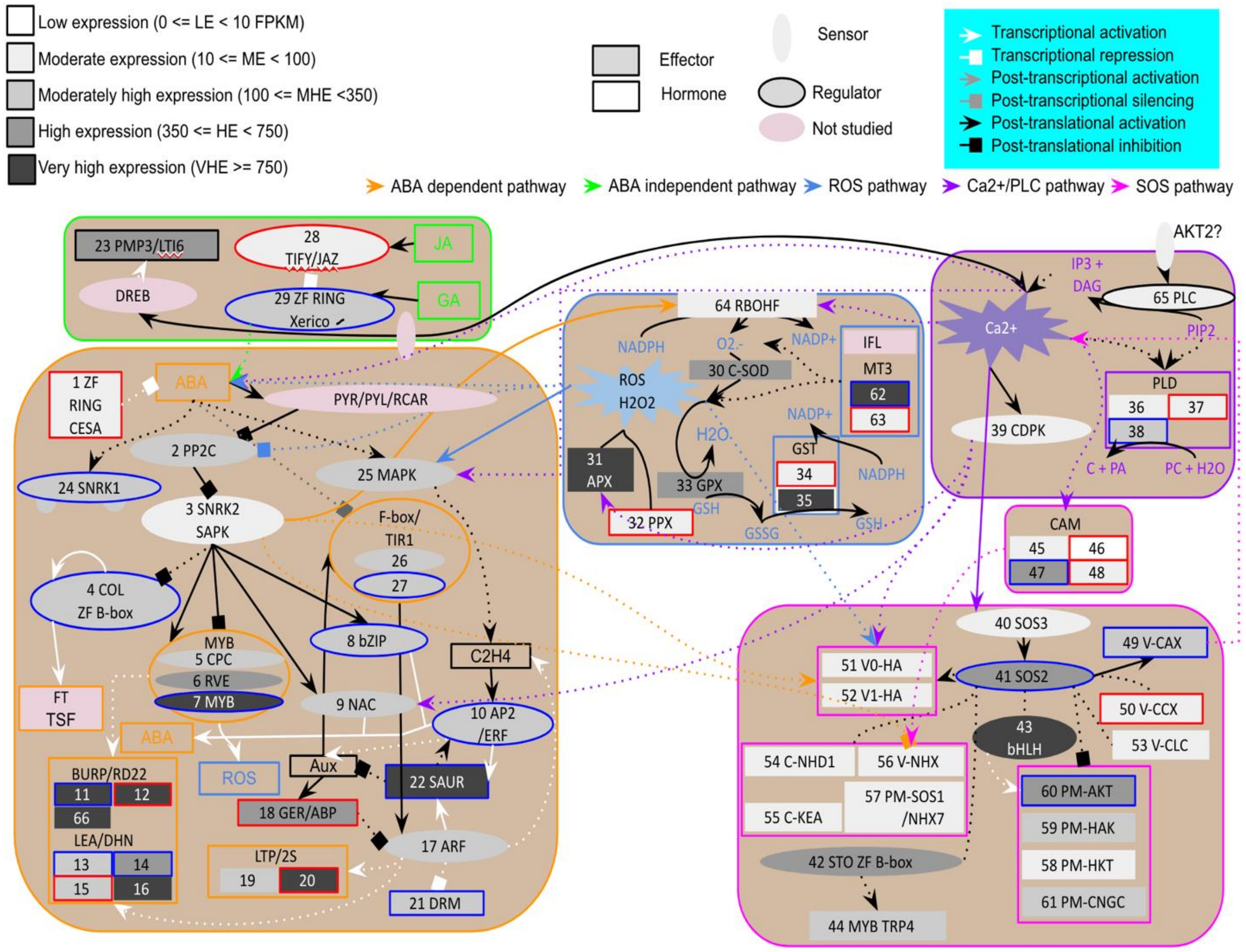

Figure S9c: 3rd animated fig: RNA-Seq expression at 10 d in coconut leaf signaling pathways during salt-water stress

Graphical conventions are the same as in figure S9a. In contrast with the early response, where 12 proteins were under-expressed and four over-expressed, this trend is reversed at 10d: 13 proteins are under-expressed ECNs against 18 over-expressed.

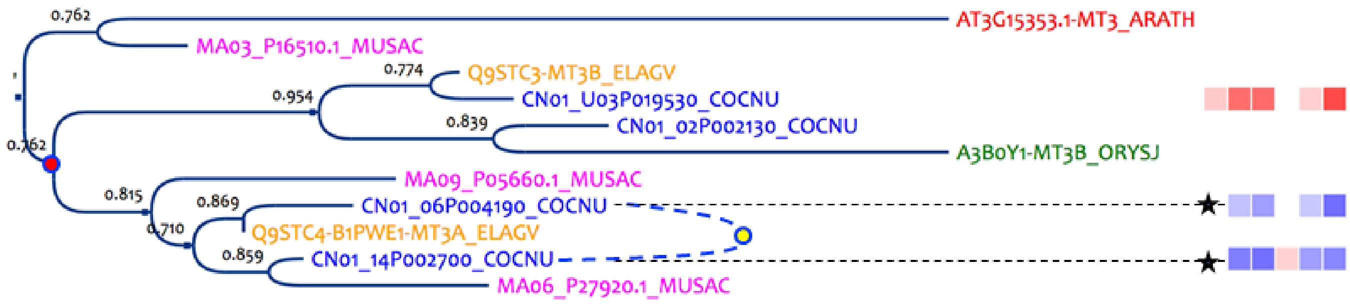

Figure S10: MT3 gene family (ROS signalling pathway)

Comparative omics of Metallothionein-like protein 3 (MT3) genes including sequences from coconut (COCNU), oil palm (ELAGA), banana (MUSAC), *japonica* rice (ORYSJ) and Arabidopsis (ARATH). No homologs were found in date palm. Colored bullets indicate WGDs (yellow for *p* WGD and red for τ WGD). Note that the topology of this dendrogram does not necessarily the true phylogeny of the genes because it was computed based on a short multiple protein alignment: only 28 out of a total of 53 aminoacids are polymorphic. We use dashed lines to represent probable paralogy relationship as inferred based on synteny groups (see figures 4 and 5, in the present case, chromosomes 6 and 14 of coconut represent block A, duplicated at WGD *p*) To the right, of the figure, expression differential is displayed with blue and red squares for up and down-regulation respectively. The first three columns are for the Tall variety (0 vs 4h, 0 vs 6d and 0 vs 10d) and the last three are for the Dwarf variety. Candidate genes, CN01_14G002700 and CN01_06G004190, are constitutively highly expressed in the leaves of this coconut individual under this salinity stress experimentation.

Figure S11: Expression of the ABA-ABAi pathway in coconut leaves under salt stress

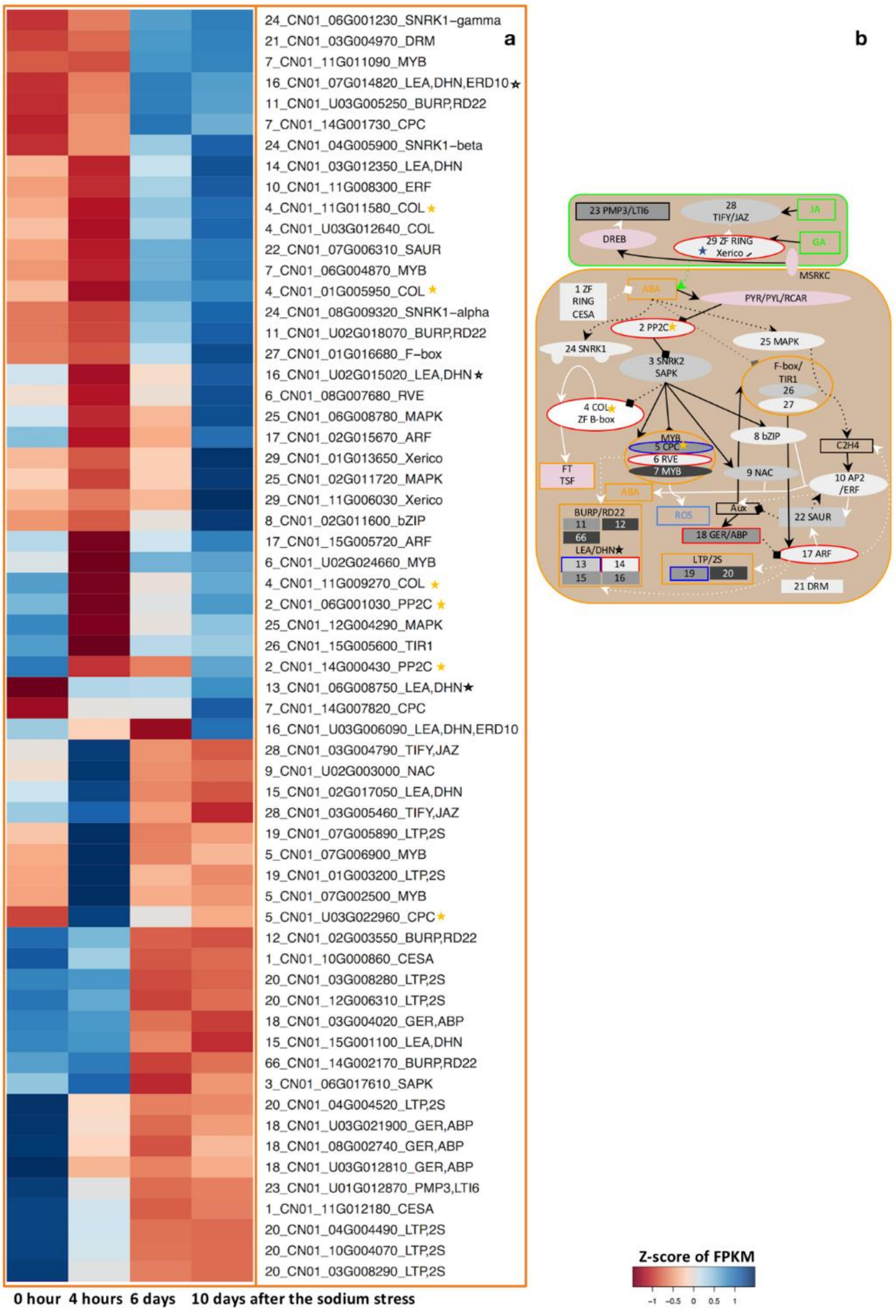

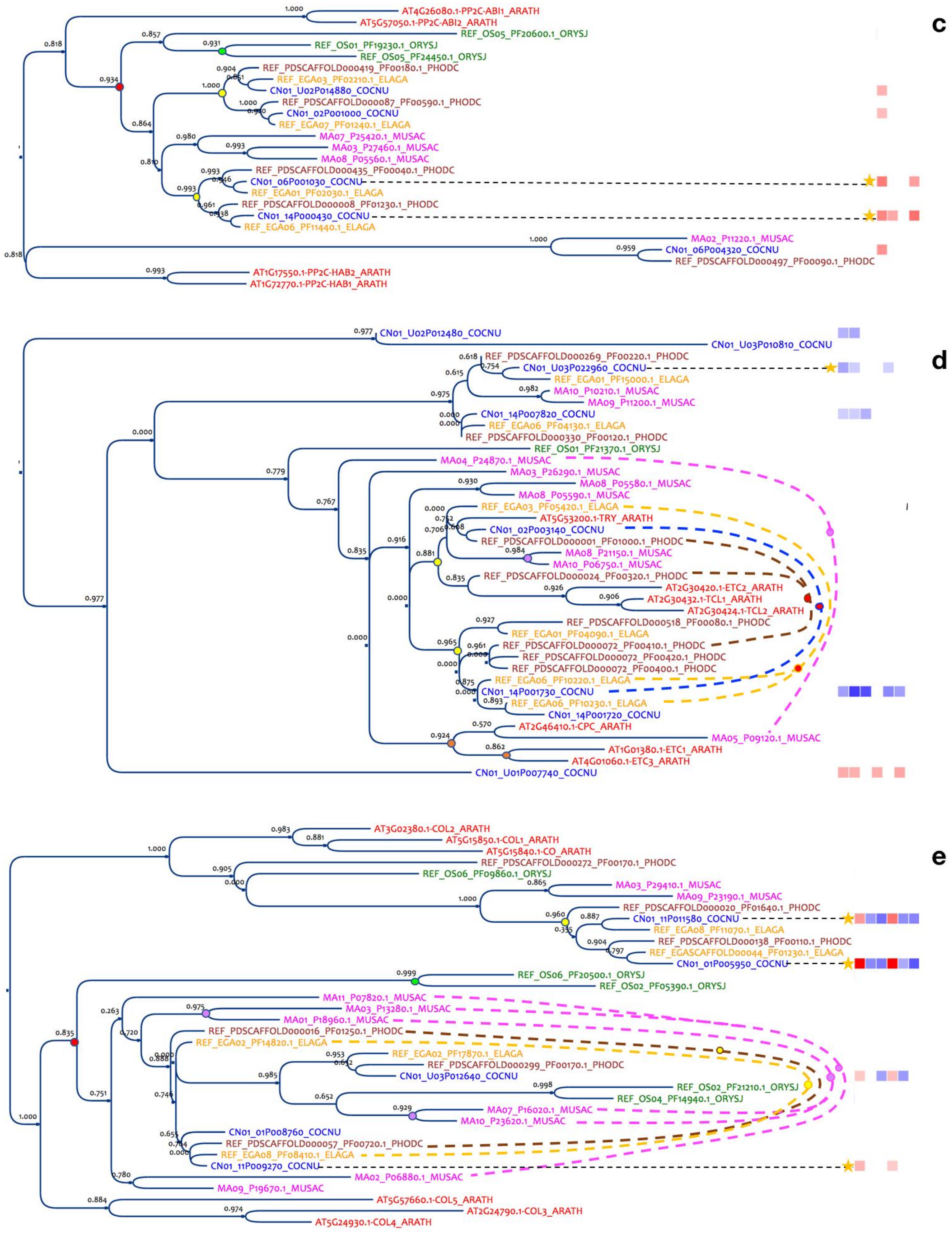

Figure S11: Expression of the ABA-ABAi pathway in coconut leaves under salt stress

a) Levels of normalized transcriptional expression in Hainan Tall leaves at time points 0, 4h, 6d and 10d after a salt stress for the DE and CHE genes of the ABA and ABAi pathways. Each gene is identified by its expression class number (ECN), gene identifier and function identifier. We assigned the same ECN to genes with similar putative functions and expression profiles.

b) Schematic representation of the ABA and ABAi signaling pathway at 4h (see legend of Figure S9).

c), d), e) Integrated comparative omics on phylogenetic trees for the PP2C, MYB and COL gene families. Colored bullet are used to represent whole genome di- or triploidisations events in monocots predicted by IDEVEN (https://github.com/Delphine-L/IDEVEN): yellow for WGDs *p*, red for τ, purple for $\alpha/\beta/\gamma$, green for ρ/σ. Orange is used for whole genome di- or triploidisations events in dicots.(see https://genomevolution.org/wiki/index.php/Plant_paleopolyploidy). Dashed lines represent probable paralogy relationships as inferred based on synteny blocks (e.g. in figure S11d, two coconut genes located on chromosomes 2 and 14 belong respectively to blocks E and A, which diverged after WGD τ - see figure 5). Expression differential is displayed with blue and red squares for up and down-regulation respectively; the first three columns are for the Hainan Tall variety (0 vs 4h, 0 vs 6d and 0 vs 10d) and the three lasts are for the Aromatic Dwarf variety.

c) case of the PP2C group A family (ECN 2). CN01_06P001030 and CN01_14P000430 are the most down-regulated Protein phosphatase 2C genes (from a moderately high to a moderate expression) in our salt stress experiment. According to the Singh *et al.*'s[14] classification and this phylogeny, the five coconut genes could belong to the sub-clade 'a' of group A *PP2C*s (ABI, HAB). This family seems well conserved over its evolutionary history: in palms, we note four sets of orthologous genes, which are likely to result from WGDs *p* and τ.

d) Case of the CPC MYB transcription factor gene family, (ECN 5, 6 and 7). CN01_14P007820 and CN01_U03P022960 are the most expressed Caprice genes (VH and H). CN01_14P001720 and CN01_14P001730 could be two tandem duplicated genes. All species in this tree, except for rice, show tandem duplications. Theses duplications appear to have occurred at the specific level in most cases, but in the case of coconut and oil palm, it seems to have occurred in their common ancestor (out-paralogs).

e) Case of the CO-COL transcription factor gene family (ECN 4). CN01_01P005950, CN01_11P009270, CN01_11P011580 and CN01_U03P012640 are similar to Zinc finger protein CONSTANS (CO) and CONSTANS-LIKE (COL). The first three genes are differentially expressed whereas CN01_U03P012640 is constitutively highly expressed. The typical expression profile is down-regulated at early stage and up-regulated at the late stages. In the two clades of this phylogenetic tree, gene retention after WGDs is observed. The paralogous coconut genes, CN01_01P005950 and CN01_11P011580 (apparently resulting from WGD p) are co-orthologs of A. thaliana of the subfamily CO, COL1, COL2 of this CONSTANS family; while CN01_01P008760, CN01_11P009270 and CN01_U03P012640 are co-orthologs of COL3, COL4 and COL5.

4. GBS processing pipeline

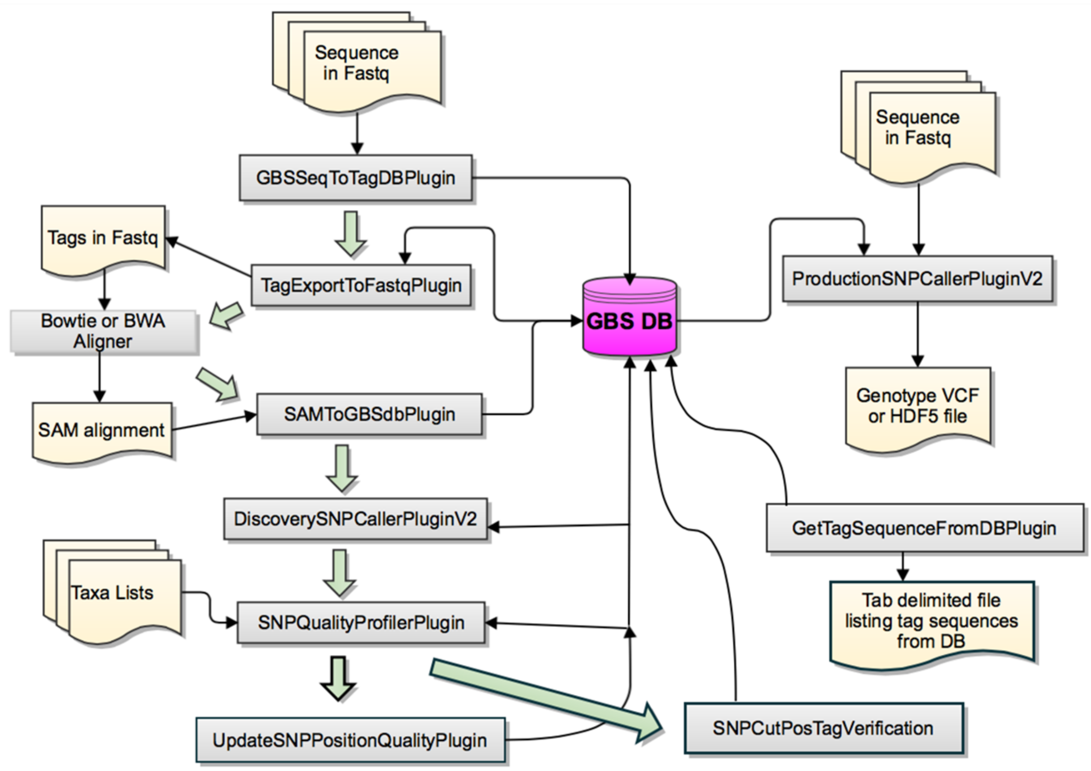

Figure S12: Tassel 5 GBS v2 Pipeline

See https://bitbucket.org/tasseladmin/tassel-5-source/wiki/Tassel5GBSv2Pipeline

Table S1: Parameters used with TASSEL

Step (plugin)	Parameters	Results	Count	%
00_raw_fq (input)		Read tot nb in lanes	1,629,747,212	
01_GBSSeqToTagDB	ePstI-MseI c5 kmerL81 minKmerL20 mnQS10	Good barcoded read tot nb	1,132,610,158	
02_TagExportToFastq	c9	Tag tot nb	3,336,897	
03_BowtieToSam	very-sensitive	Tags aligned =1 time	2,224,679	66.67
03_BowtieToSam	very-sensitive	Tags aligned >1 times	520,656	15.6
04_SAMToGBSdb	aProp0 aLen0	Mapped tag tot nb	2,745,335	82.27
05_DiscoverySNPCaller	maxTagsCutSite81 mnLCov0.1 mnMAF0.01	SNP tot nb	1,792,810	
05_DiscoverySNPCaller		Allele tot nb	3,746,887	
06_SNPQualityProfiler			1,792,810	
07_ProdSNPCaller (VCF)	ePstI-MseI kmerL81 mnQS10		3,746,887	

Table S2: Parameters used with Process reseq

Step	Parameters	Result
1: demultiplex	met4c rePstI	GBS1-6
2: cutadapt	aTruSeq_adapter_RC.fasta m20	GBS1-6
3: filter_fq_mean_qual	a33 m30 l35	GBS1-6
4a: process_reseq BWA mapping per ind.	242 ind_GBS_clean.fastq	BAM file nb
	368 missing_long_scaffold.fna t50	
4c: PR realign per ind.	t274	Realigned BAM file nb
4e: PR SNP calling	t4	GVCF file nb
4f: PR VCF creation	t4	Marker nb
5: VCFprefilter	m5 M1000 f0.01 c2	Marker nb
6: VCFfilter	m5 M1000 f0.01:0.1 c1 s0.8 p1e-20	Scaffold nb, Marker nb

Table S3: Parameters used with VCF filter

Step	Parameters	Results	Count
08_MonomorphicFilter	Alt allele "."	Monomorphic SNP tot nb	1,161,527
08_MonomorphicFilter	Ref allele "N"	Gap tot nb	16,091
08_MonomorphicFilter		SNP tot nb after MF	631,283
09_VCFFilter	WinFreq0.01:0.1 miss0.5 pValue1e-20	SNP tot nb after VCFF	17,548

Table S4: Parameters used with Tassel_to_J and Joinmap

Step	Parameters
01_R Creation of Genotypic & marker info files	PvalueMin 1e-20
02_R Filtering makers according to their parents	missInd 0.5<br>MAF 0.15<br>fewGoodAllele 0.7<br>manyWrongAllele 0.1
03_R Filtering makers according to disjonctions	genotypeCode ABHU
04_R Filtering makers according to missing data per marker	tOutlier 60<br>lGap 0.05<br>majority 0.8<br>stopRemove 20<br>maxMissLoc0.2
05_R write the final SNP matrix file for JoinMap	rmNbInd 0
Parameters for Joinmap	Pop.type:bc1
	Nrofloci: 8466
	Nrofind: 2317

Table S5: Parameters used with Scaffhunter

Step	Parameters	Result	Outcome
1a: Scaffhunter JMpwd2matrix.py	rec lod	2 distance matrix files per chr	16 LOD.mat
			16 REC.mat
		Marker nb	8465
		Scaffold nb	2316
1c: SH Ordering of scaffolds using an UPGMA like method	Type IDENT	1 scaffold order file per chr	16 LOD_scaff_order.tab
		1 marker order along scaffold file per chr	16 LOD_mark_order.tab
1d: SH reorderient Optimization of scaffold order and orientation	Type IDENT	1 reoriented scaffold order file per chr	16 LOD_scaff_order_opt.tab
		1 marker order along scaffold file per chr	16 LOD_mark_order_opt.tab
1e: SH matrix2ortho	Type LOD	1 dotplot file per chr before optimization	16 LOD_mark_linkage_before.png
Markers linkage visualization by dotplot		1 dotplot file per chr after optimization	16 LOD_mark_linkage_after.png
1f: SH scaff2chrom		Marker nb	8402
		Scaffold nb	2303
		Agp file<br>Pseudomolecule fasta file	16 chromosomes<br>chrUn_random

5. RNA-Seq analysis bioinformatics pipeline

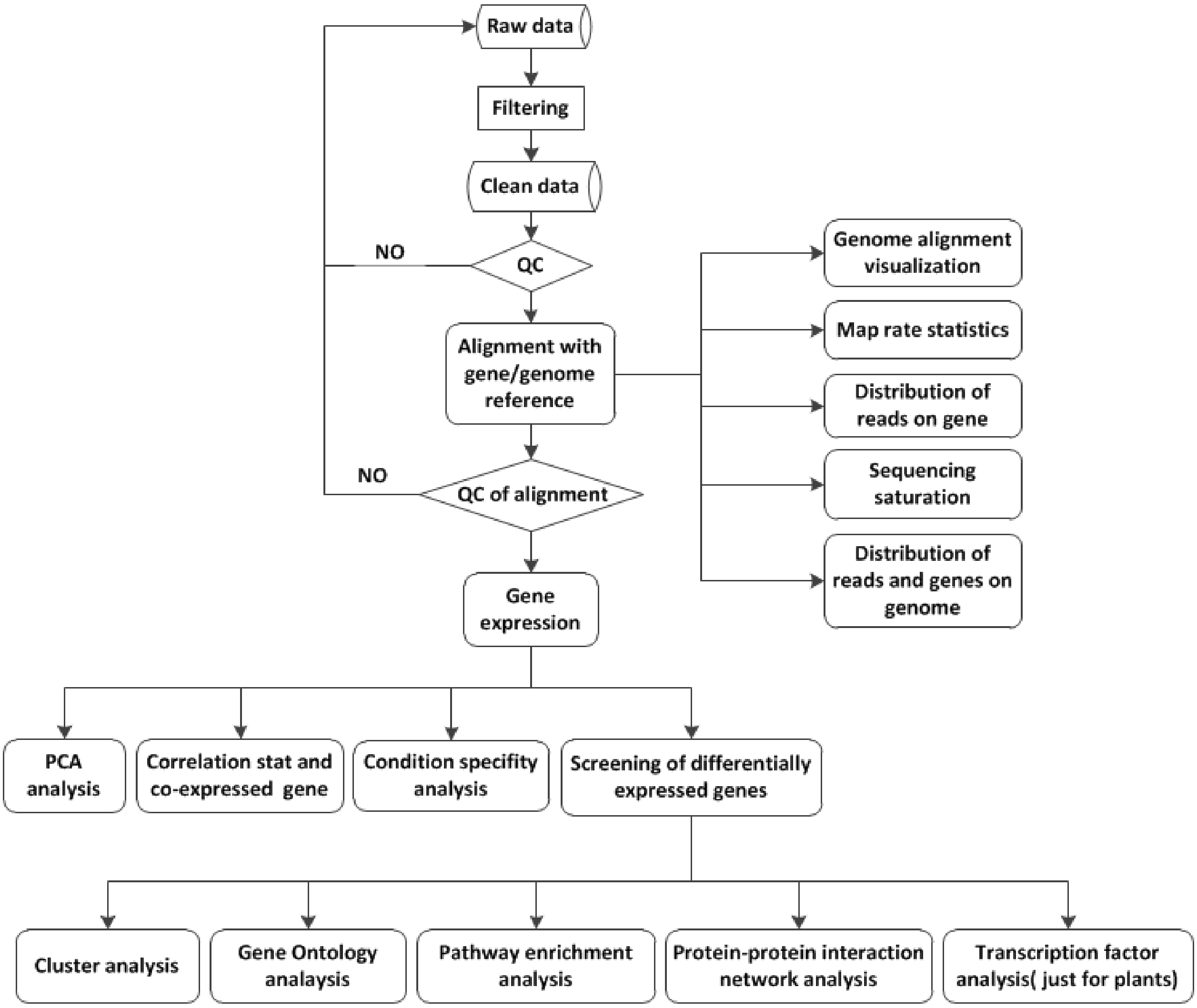

Figure S13: RNA-Seq analysis bioinformatics workflow

Primary sequencing data that produced by Illumina HiSeq^{TM} 4000, called as raw reads, is subjected to quality control (QC) that determine if a resequencing step is needed. After QC, raw reads are filtered into clean reads which will be aligned to the reference sequences (BWA for genome reference and Bowtie for gene reference). QC of alignment is performed to determine if resequencing is needed. The alignment data is utilized to calculate distribution of reads on reference genes and mapping ratio. If alignment result passes QC, we will proceed with downstream analysis including gene expression and deep analysis based on gene expression (PCA/correlation/screening differentially expressed genes with RSEM software package and so on). Further, we also can perform deep analysis based on DEGs, including Gene Ontology (GO) enrichment analysis, Pathway enrichment analysis, cluster analysis, protein-protein interaction network analysis and finding transcription factor.

6. REFERENCES

1. Soda, N., Sharan, A., Gupta, B.K., Singla-Pareek, S.L. & Pareek, A. Evidence for nuclear interaction of a cytoskeleton protein (OsIFL) with metallothionein and its role in salinity stress tolerance. *Sci Rep* **6**, 34762 (2016).
2. Taji, T. et al. Comparative genomics in salt tolerance between *Arabidopsis* and Arabidopsis-related halophyte salt cress using *Arabidopsis* microarray. *Plant Physiol* **135**, 1697-709 (2004).
3. Rozema, J. & Schat, H. Salt tolerance of halophytes, research questions reviewed in the perspective of saline agriculture. *Environmental and Experimental Botany* **92**, 83-95 (2013).
4. Kumar, K., Kumar, M., Kim, S.R., Ryu, H. & Cho, Y.G. Insights into genomics of salt stress response in rice. *Rice (N Y)* **6**, 27 (2013).
5. Golldack, D., Li, C., Mohan, H. & Probst, N. Tolerance to drought and salt stress in plants: Unraveling the signaling networks. *Front Plant Sci* **5**, 151 (2014).
6. Julkowska, M.M. & Testerink, C. Tuning plant signaling and growth to survive salt. *Trends Plant Sci* **20**, 586-94 (2015).
7. Tao, J.J. et al. The Role of Ethylene in Plants Under Salinity Stress. *Front Plant Sci* **6**, 1059 (2015).
8. Sah, S.K., Reddy, K.R. & Li, J. Abscisic Acid and Abiotic Stress Tolerance in Crop Plants. *Front Plant Sci* **7**, 571 (2016).
9. Hua, D. et al. A plasma membrane receptor kinase, GHR1, mediates abscisic acid- and hydrogen peroxide-regulated stomatal movement in Arabidopsis. *Plant Cell* **24**, 2546-61 (2012).
10. Bose, J., Rodrigo-Moreno, A. & Shabala, S. ROS homeostasis in halophytes in the context of salinity stress tolerance. *J Exp Bot* **65**, 1241-57 (2014).
11. Maathuis, F.J. Sodium in plants: perception, signalling, and regulation of sodium fluxes. *J Exp Bot* **65**, 849-58 (2014).
12. Zhang, Z., Rosenhouse-Dantsker, A., Tang, Q.Y., Noskov, S. & Logothetis, D.E. The RCK2 domain uses a coordination site present in Kir channels to confer sodium sensitivity to Slo2.2 channels. *J Neurosci* **30**, 7554-62 (2010).
13. Cuellar, T. et al. Potassium transport in developing fleshy fruits: the grapevine inward K(+) channel VvK1.2 is activated by CIPK-CBL complexes and induced in ripening berry flesh cells. *Plant J* **73**, 1006-18 (2013).
14. Singh, A., Jha, S.K., Bagri, J. & Pandey, G.K. ABA inducible rice protein phosphatase 2C confers ABA insensitivity and abiotic stress tolerance in Arabidopsis. *PLoS One* **10**, e0125168 (2015).
