# Supplementary material for: Coconut genome assembly enables evolutionary analysis of palms and highlights signaling pathways involved in salt tolerance
Source: Commun Biol. 2021 Jan 22;4:105. doi: 10.1038/s42003-020-01593-x (PMC7822834; doi:10.1038/s42003-020-01593-x)
Supplement: Supplementary file 3 — Description of Additional Supplementary Files [file 42003_2020_1593_MOESM3_ESM.pdf]

Description of Additional Supplementary Files

File name: Supplementary Data 1
Description: 2666 paralogous coconut gene models.

Column #	Title	Comment
1	IdGs	Identity of the paralogous group (synteny block)
information on gene 1		
2	Gen	Gene identifier
3	Chromosome	Chromosome number
4	PosI	Starting point of gene
5	PosF	Ending point of gene
information on gene 2		
6	Gen	Gene identifier
7	Chromosome	Chromosome number
8	PosI	Starting point of gene
9	PosF	Ending point of gene

Example

IdGs	Gen	Chromosome	PosI	PosF	Gen	Chromosome	PosI	PosF
Gs.1	CN01_12G003260	CN01_12	26778862	26783317	CN01_01G000810	CN01_01	1913418	1918873
Gs.1	CN01_12G003240	CN01_12	26746589	26747020	CN01_01G000830	CN01_01	1938309	1939216
Gs.1	CN01_12G003210	CN01_12	26655195	26675707	CN01_01G000880	CN01_01	2037000	2051243

[…]

File name: Supplementary Data 2
Description: 9866 orthologous gene pairs in coconut and oil palm.

Column	comment
1	Coconut gene identifier
2	Coconut chromosome number
3	Starting point of coconut gene
4	Ending point of coconut gene
5	Oil palm identifier
6	oil palm chromosome number
7	Starting point of oil palm gene
8	Ending point of oil palm gene

Example

CN01_01G003840	1	9416291	9418351	XP_010914194.1	2	55227519	55230001
CN01_01G003830	1	9394065	9395304	XP_010914193.1	2	55207874	55209272
CN01_01G003820	1	9384543	9390831	XP_010914191.1	2	55197200	55204811

[…]

File name: Supplementary Data 3 – 8
Description: An Excel file which contains the following tables

Supplementary Data 3: ***Gap in Cn15****:* a tentative to fill in the gap resulting from the presence of a gene involved in pollen selection in the middle part of Cn15. Protein genes in these scaffolds are orthologous to genes located in the corresponding portion of chromosome 9 of *Elaeis guineensis*.

Supplementary Data 4: ***RNA-seq on genome****:* alignment statistics result of clean RNA-Seq reads HAIT reference genome for all *C. nucifera* leaf samples of the salt stress experiment.

Supplementary Data 5: ***RNAseq on genes:*** alignment statistics result of clean RNA-Seq reads against HAIT reference genes for all *C. nucifera* leaf samples of the salt stress experiment.

Supplementary Data 6: ***DE & CHE genes:*** *Cocos nucifera* differentially expressed (590) or constitutively highly expressed genes (194) during the salt stress experiment.

Supplementary Data 7: ***Salt related genes:*** 152 *Cocos nucifera* genes which are ortholog to genes known to be involved in salt stress response in other plant species.

Supplementary Data 8: ***Common genes:*** 65 *Cocos nucifera* genes, which are common to Supplementary Data 6 and 7.
