# Supplementary material for: Coconut genome assembly enables evolutionary analysis of palms and highlights signaling pathways involved in salt tolerance
Source: Commun Biol. 2021 Jan 22;4:105. doi: 10.1038/s42003-020-01593-x (PMC7822834; doi:10.1038/s42003-020-01593-x)
Supplement: Supplementary file 7 — Reporting Summary [file 42003_2020_1593_MOESM7_ESM.pdf]

nature research

Corresponding author(s): Luc Baudouin

Last updated by author(s): Aug 5, 2020

Reporting Summary

Nature Research wishes to improve the reproducibility of the work that we publish. This form provides structure for consistency and transparency in reporting. For further information on Nature Research policies, see our Editorial Policies and the Editorial Policy Checklist.

Statistics

For all statistical analyses, confirm that the following items are present in the figure legend, table legend, main text, or Methods section.

n/a	Confirmed	
☐	☒	The exact sample size (*n*) for each experimental group/condition, given as a discrete number and unit of measurement
☐	☒	A statement on whether measurements were taken from distinct samples or whether the same sample was measured repeatedly
☒	☐	The statistical test(s) used AND whether they are one- or two-sided<br>*Only common tests should be described solely by name; describe more complex techniques in the Methods section.*
☒	☐	A description of all covariates tested
☒	☐	A description of any assumptions or corrections, such as tests of normality and adjustment for multiple comparisons
☐	☒	A full description of the statistical parameters including central tendency (e.g. means) or other basic estimates (e.g. regression coefficient) AND variation (e.g. standard deviation) or associated estimates of uncertainty (e.g. confidence intervals)
☒	☐	For null hypothesis testing, the test statistic (e.g. *F*, *t*, *r*) with confidence intervals, effect sizes, degrees of freedom and *P* value noted<br>*Give P values as exact values whenever suitable.*
☒	☐	For Bayesian analysis, information on the choice of priors and Markov chain Monte Carlo settings
☒	☐	For hierarchical and complex designs, identification of the appropriate level for tests and full reporting of outcomes
☒	☐	Estimates of effect sizes (e.g. Cohen's *d*, Pearson's *r*), indicating how they were calculated

Our web collection on statistics for biologists contains articles on many of the points above.

Software and code

Policy information about availability of computer code

Data collection	Mapping population:<br>* TASSEL-GBS20 pipeline V5.2.29 to locate SNPs on scaffolds.<br>* vcf2pop filters VCF to select usable SNPs https://github.com/SouthGreenPlatform/VcfHunter.<br>* In house 'Tassel_to_J ' pipeline in R Markdown available at https://github.com/SouthGreenPlatform/Curation-GBS-data to construct the data matrix after further filtering.<br><br>RNA-Seq data:<br>* BWA to map clean reads to genome reference.<br>* Bowtie to map clean reads to gene reference.<br>* RSEM package to quantify gene expression.<br>* Noiseq to identify differentially expressed genes.
Data analysis	Mapping population:<br>* Joinmap to identify linkage groups.<br>* Scaffhunter to find scaffold order and orientation within linkage groups.<br>* In house mapping procedure available at https://github.com/SouthGreenPlatform/Curation-GBS-data to compute linkage distances in presence of genotyping errors.<br>* In house optimization procedure available at vailable at https://github.com/SouthGreenPlatform/Curation-GBS-data to locate a gene involved in pollen-stigma interaction and evaluate selection pressure.

LTR Retrotransposon distribution over insertion time:
* MUSCLE to align LTR retrotransposon sequences.
* EMBOSS to compute genetic distance between LTR sequences.

For manuscripts utilizing custom algorithms or software that are central to the research but not yet described in published literature, software must be made available to editors and reviewers. We strongly encourage code deposition in a community repository (e.g. GitHub). See the Nature Research guidelines for submitting code & software for further information.

Data

Policy information about availability of data

All manuscripts must include a data availability statement. This statement should provide the following information, where applicable:
- Accession codes, unique identifiers, or web links for publicly available datasets
- A list of figures that have associated raw data
- A description of any restrictions on data availability

The final assembly and annotation are deposited in DDBJ/EMBL/GenBank under the following identifiers: SUBID: SUB5865736, BioProject: PRJNA374600, BioSample: SAMN06328965, Accession: VOII00000000, Organism Cocos nucifera. Genetic map, genome sequence and annotation can be obtained and viewed at http://palm-genome-hub.southgreen.fr/. Transcriptomic data (RNA-Seq and differential expression) are available under GEO Superseries Accession GSE134410. Mapping population GBS data are available under BioSample accessions: SAMN15659886 to SAMN15660159 and SRA runs: 15085388 to 15085437.

Field-specific reporting

Please select the one below that is the best fit for your research. If you are not sure, read the appropriate sections before making your selection.

☒ Life sciences ☐ Behavioural & social sciences ☐ Ecological, evolutionary & environmental sciences

For a reference copy of the document with all sections, see nature.com/documents/nr-reporting-summary-flat.pdf

Life sciences study design

All studies must disclose on these points even when the disclosure is negative.

Sample size	Mapping population: 320 individuals of a mapping population were produced by controlled hand pollination, 216 of them were used for cartography.<br>Response to salt stress: Three seedlings of Hainan Tall and three seedlings of Aromatic Dwarf.
Data exclusions	Mapping population: 28 individuals discarded based on a legitimacy tests using microsatellite markers, 52 based on sequencing statistics, 22 based on insufficient number of markers.
Replication	Mapping population: Not applicable.<br>Response to salt stress: One leaf samples were collected from three individual of each variety at each sampling time (Some were missing at 10 days).
Randomization	Not applicable
Blinding	Not applicable

Reporting for specific materials, systems and methods

We require information from authors about some types of materials, experimental systems and methods used in many studies. Here, indicate whether each material, system or method listed is relevant to your study. If you are not sure if a list item applies to your research, read the appropriate section before selecting a response.

Materials & experimental systems

n/a	Involved in the study	
☒	☐	Antibodies
☒	☐	Eukaryotic cell lines
☒	☐	Palaeontology and archaeology
☒	☐	Animals and other organisms
☒	☐	Human research participants
☒	☐	Clinical data
☒	☐	Dual use research of concern

Methods

n/a	Involved in the study	
☒	☐	ChIP-seq
☒	☐	Flow cytometry
☒	☐	MRI-based neuroimaging
